# Abnormal Sleep Signals Vulnerability to Chronic Social Defeat Stress

**DOI:** 10.3389/fnins.2020.610655

**Published:** 2021-01-12

**Authors:** Basma Radwan, Gloria Jansen, Dipesh Chaudhury

**Affiliations:** ^1^Department of Biology, New York University Abu Dhabi, Abu Dhabi, United Arab Emirates; ^2^Department of Genetics, University of Cambridge, Cambridge, United Kingdom

**Keywords:** depression, EEG, NREM sleep, fragmentation, insomnia, chronic social defeat

## Abstract

There is a tight association between mood and sleep as disrupted sleep is a core feature of many mood disorders. The paucity in available animal models for investigating the role of sleep in the etiopathogenesis of depression-like behaviors led us to investigate whether prior sleep disturbances can predict susceptibility to future stress. Hence, we assessed sleep before and after chronic social defeat (CSD) stress. The social behavior of the mice *post stress* was classified in two main phenotypes: mice susceptible to stress that displayed social avoidance and mice resilient to stress. Pre-CSD, mice susceptible to stress displayed increased fragmentation of Non-Rapid Eye Movement (NREM) sleep, due to increased switching between NREM and wake and shorter average duration of NREM bouts, relative to mice resilient to stress. Logistic regression analysis showed that the pre-CSD sleep features from both phenotypes were separable enough to allow prediction of susceptibility to stress with >80% accuracy. Post-CSD, susceptible mice maintained high NREM fragmentation while resilient mice exhibited high NREM fragmentation, only in the dark. Our findings emphasize the putative role of fragmented NREM sleep in signaling vulnerability to stress.

## Introduction

Global sleep problems are on the rise and negatively impact the quality of life and health, leading to chronic diseases including mood disorders. Common life stresses lead to sleep impairments such as reduced sleep time, compromised sleep consolidation and increased REM intensity (Becker-Carus and Heyden, 1979; Edell-Gustafsson and Hetta, 1999; Harvey et al., 2005; Akerstedt et al., 2007). Moreover, sleep impairments are among the core symptoms of many stress-induced psychiatric disorders such as depression and anxiety (Hefti et al., 2013; Difrancesco et al., 2019). Among individuals suffering from depression, insomnia and hypersomnia are the most prevalent sleep disorders (Nutt et al., 2008) where common symptoms that affect sleep quality include prolonged sleep latency, difficulty maintaining sleep and early morning awakenings (Steiger and Kimura, 2010). These observations highlight the complex inter-relationship between mood and sleep as sleep disturbances are both symptoms of and a risk factor for mood disorders.

Sleep is a complex biological function that induces neural plastic changes in the brain (de Vivo et al., 2017; Eban-Rothschild et al., 2017). According to the Synaptic Homeostasis Hypothesis (SHY) (Vyazovskiy et al., 2008; Tononi and Cirelli, 2012), sleep re-establishes synaptic homeostasis via renormalization of the synapses following net potentiation during wake. For example, some evidence suggests that slow wave activity (SWA) leads to synaptic downscaling (Tononi and Cirelli, 2003). Thus, sleep is hypothesized to “recalibrate” synaptic plasticity, which is believed to be the neural basis for adaptive behavior (Goldstein and Walker, 2014; Kuhn et al., 2016). Consequently, sleep disturbances related to psychiatric disorders are conceptualized to be a maladaptive response to stress, while impaired sleep prior to stress may compromise the brain’s ability to adapt to the stressor (Radwan et al., 2019; Cheng et al., 2020). A growing body of evidence suggests that Rapid Eye Movement (REM) or Non-Rapid Eye Movement (NREM) sleep disturbances can be used as markers of vulnerability to psychiatric disorders such as anxiety, depression and post-traumatic stress disorder (PTSD) (Thase et al., 1998; Rao et al., 2009; Steiger and Pawlowski, 2019). It is postulated that disturbed sleep prior to a stressor might lead to subsequent mood disorders as it may contribute to the imbalance between excitation and inhibition or impairment of the restorative function of sleep required to adapt to stress (Bryant et al., 2010; Parrino and Vaudano, 2018). Indeed, insomnia is a strong risk factor for depression as it commonly precedes development of mood disorders (Ford and Kamerow, 1989; Breslau et al., 1996; Chang et al., 1997; Perlis et al., 1997; Franzen and Buysse, 2008; Batterham et al., 2012, 2017; Fernandez-Mendoza et al., 2016; Li et al., 2016; Goldschmied et al., 2019).

Repeated exposure to social stress, during the chronic social defeat (CSD) stress paradigm, is a commonly used rodent model of stress as it leads to continuous activation of the hypothalamic-pituitary-adrenal (HPA) axis and induction of depressive-like phenotypes such as social avoidance, anhedonia and anxiety (Krishnan et al., 2007; Krishnan and Nestler, 2011; Chaudhury et al., 2013). Chronic stress exposure leads to two behavioral phenotypes: “stress-susceptible”-mice that display low social interaction (SI) scores and “stress-resilient”-mice that display high SI scores. Interestingly, stress-susceptible and stress-resilient mice exhibit distinct molecular mechanisms, circuit changes and synaptic reorganization (Krishnan et al., 2007; Chaudhury et al., 2013, 2015). Importantly, the social avoidance behaviors last for few weeks post CSD stress (Berton et al., 2006; Krishnan et al., 2007). Though a few studies have examined the effect of CSD on sleep (Henderson et al., 2017; Olini et al., 2017; Wells et al., 2017; Fujii et al., 2019), to date, no study has explored the characteristics of sleep of the stress-susceptible versus the stress-resilient mice *prior* to CSD. Thus, we examined sleep in adult C57BL/6J male mice pre- and post-exposure to a 15-d CSD paradigm. Moreover, homeostatic sleep response following 4-h sleep deprivation (SD) was also assessed pre- and post-exposure to chronic social stress. Our findings demonstrate that mice that become susceptible to CSD stress exhibit *pre-existing* abnormal sleep/wake characteristics prior to stress exposure. Moreover, subsequent exposure to stress further impairs sleep and the homeostatic response.

## Materials and Methods

### Ethics Statement

All experiments performed were approved by the NYUAD Animal Care and Use Committee, and all experimental protocols were conducted according to the National Institute of Health Guide for Care and Use of Laboratory Animals (IACUC Protocol: 150005A2).

### Animals

CD1 retired breeders (Charles River, United Kingdom), and C57BL/6J male mice (10–16 weeks; Jackson Laboratories, ME, United States) were used in this study. All mice were maintained in the home cages, with *ad libitum* access to food and water in temperature (21 ± 2°C)- and humidity (50 ± 10%)-controlled facilities with 12-h light-dark (L/D) cycles (lights on at 7:00 AM and lights off at 7:00 PM, zeitgeber time (ZT 0 = lights on, ZT 12 = light off). Zeitgeber time is a unit of time based on 12:12 light: dark cycle. All behavioral experiments were conducted during the light cycle (ZT 5 to ZT 10).

### Chronic Social Defeat Stress Paradigm

Chronic social defeat stress paradigm was performed according to previously published protocols (Krishnan et al., 2007; Golden et al., 2011; Chaudhury et al., 2013). The CD1 mice were screened upon arrival and the aggressive ones were selected. CD1 aggressor mice were single housed to habituate at least 48–72 h on one side of a clear perforated plexiglass divider. Experimental C57BL/6J mice were introduced into the cage of a novel and aggressive CD1 mouse for 10-min during which time they were physically attacked by the CD1 mouse. After 10-min of physical contact, the C57BL/6J mice were separated by a clear perforated plexiglass divider for the following 24-h allowing sensory, but not physical contact. The social defeat stress was repeated for 15-d for each C57BL/6J mouse, using a novel aggressor daily. We chose the 15-d protocol of CSD to ensure the chronicity of stress and its potential impact on sleep and wake. The stress-naïve/control mice were housed in pairs within a cage continuously separated by a clear perforated plexiglass divider. Additionally, the stress-naïve mice were moved daily to a different room for the duration of the defeat (10 min) to eliminate the passive stress effect. On the last day of defeat (Day 15), the mice were singly housed in new cages. The current study was conducted across a total of 8 blocks, each containing 2–4 mice in a staggered approach following the same timeline. CD1 mice were screened for frequency and latency for attacks before each CSD such that those mice that exhibited similar attack latencies of equal to or less than 30-s with comparable number of attacks were used. This ensured that the C57BL/6J mice were exposed to similar level of aggression. Additionally, all C57BL/6J used in this study started around the same age (10 ± 2 weeks).

### Social Interaction Test

On recovery day 16, social-avoidance behavior toward a novel non-aggressive but active CD1 mouse was measured in a two-trial social-interaction test. In the first 2.5 min trial, the experimental mouse was allowed to freely explore a square-shaped arena (44 cm × 44 cm) containing a perforated plexiglass cage (10 cm × 6 cm) along on one side of the arena (“No target” condition). In the second 2.5 min trial, the experimental mouse was reintroduced back into the arena with an unfamiliar CD1 non-aggressive mouse placed in the plexiglass cage (“Target” condition). Between trials, the behavioral apparatus was cleaned with MB-10 solution (Quip Laboratories, Inc., United States) to avoid persistence of olfactory cues. TopScan video tracking system (CleverSys. Inc.) was used to automatically monitor and record the amount of time the experimental mouse spent in the “interaction zone” (14 cm × 26 cm) interacting with the unfamiliar CD1 mouse, “corner zone” (10 cm × 10 cm) and “total travel” within the arena for the duration in both trials. Interaction zone time, corner zone time, total distance traveled were collected and analyzed. The classification of susceptible and resilient mice was based on the SI ratio, which was calculated as [100 × (time spent in the interaction zone during social target session)/(time spent in the interaction zone during no social target session)] as described previously (Krishnan et al., 2007; Golden et al., 2011; Chaudhury et al., 2013). All mice with scores < (100 – threshold) were classified as “susceptible” and those with scores ≥ (100 + threshold) were classified as “resilient.” Threshold was used to avoid using mice with a score close to 100. We set a threshold of 1.0%, which led us to exclude one mouse with a SI score 100.95.

### Surgery and Electrode Implantation

The animals were anesthetized with a mixture of ketamine (100 mg kg^–1^) and xylazine (10 mg kg^–1^) intraperitoneally. The mice were fixated in a stereotactic frame (Kopf instruments) at a sufficient level of anesthesia. The head was shaved, and the scalp was opened medially and the periosteum was removed. We used a dental precision driller (Stoelting) to drill four holes into the skull. The EEG electrodes were placed in the left and right part of the parietal lobe (from Bregma/caudal: −2 mm, medio-lateral: ±1.5 mm) and the right frontal lobe (from Bregma/rostral: +1 mm, medio-lateral: ±1 mm) and the grounding/reference electrode was placed in the cerebellum. Two EMG electrodes, gold plated, were lowered bilaterally into the neck muscle, directly caudal to the occipital bone. All EEG recording electrodes consisted of stainless-steel screws (Bilaney) with the following dimensions: head diameter 2.5 mm, shaft diameter: 1.57 mm, shaft length: 1.6 mm. All wires were connected to a head connector (MS 363 Pedestal PlasticsOne), which was secured over the skull using acrylic C and B Metabond (Parkell Inc.). Next, dental cement (Stoelting) was applied around the head connected to protect all the wires and the connector.

### Timeline of EEG Sleep Recording

After a minimum duration of 7 days of postoperative recovery, mice were allowed to habituate (Hab) to the sleep chambers (Viewpoint, Lyon France) for 48-h after which, 24-h baseline (BL) EEG and EMG were recorded. Moreover, the pre-CSD homeostatic response following 4-h of SD was recorded for 21-h. After 15-d of CSD and SI test, EEG and EMG baseline (BL) recordings for 24-h were performed after 48-h of Hab in the sleep chambers. Additionally, the post-CSD homeostatic response following 4-h of SD was recorded for 21-h.

### Electroencephalogram (EEG)/Electromyogram (EMG) Recording

After the postoperative recovery period, mice were transferred to a quasi-soundproof isolation sleep chamber (Viewpoint, Lyon France) under the standard laboratory conditions (12/12 h light-dark cycle, lights on at 7 am, 21 ± 2°C). Mice were connected to a cable plugged to a rotating commutator (SL-89-Opt-6, Dragonfly, Ridgeley, WV, United States) to allow free movement in all three dimensions during the chronic recording sessions (video monitored). Unipolar EEG and bipolar EMG signals were amplified 800× (TBSI, part of HBIO, Cambridge, MA, United States). The digitization was performed using a DAQ card (TBSI, part of HBIO, Cambridge, MA, United States). The data were sampled at 30 kHz. The videos were synchronized with the EEG recording via an output TTL signal (5V pulse) to trigger the start and the end of the video.

### Preprocessing, Visualization

The electrophysiological signals were filtered with a low-pass filter (cutoff frequency 7 kHz) and subsampled at 250 Hz. Next, the electrophysiological signals, the actimetry and the video were imported into a custom software program (SleepScore, Viewpoint, Lyon, France).

### Scoring of the Vigilance States

The vigilance states Wake, NREM sleep and REM sleep were visually scored off-line using the EEG and EMG signals according to standard criteria and methods (Franken et al., 1991) with a 5-s scoring window using custom software (SleepScore, Viewpoint, Lyon France). Sleep and wake states were visually analyzed and scored by the first author. The analysis was performed blind to eliminate experimenter’s bias. The occurrence of artifacts was very low (∼1–3%) in all of the mice used in the study and concentrated primarily in the wake states (motion-related artifacts). Sleep epochs containing artifacts were excluded from the spectral analysis.

### Data Analysis

The percent time, average bout duration and the number of bouts were extracted to quantitatively assess the vigilance states. Additionally, the spectral parameters of the EEG signal from right parietal electrode [e.g., power spectral density (PSD)] were computed using custom software (SleepScore, Viewpoint, Lyon France) using the mean spectrum analysis with rectangular windows with 20% overlap. Further analysis of the data was performed using scripts in Python.

### Sleep Deprivation

The 4-h SD was performed on the mice using the Viewpoint platform system at 7 AM (ZT 0). For this study, we used the basic SD paradigm consisting of sending random electric pulses in a randomized sequence to the magnet placed under the platform which pushes it up and wakes up the mice. The duration of a pulse is 1000 ms. The number of pulses per sequence is random between 3 and 5 pulses. The sequences of pulses were separated by a randomized time from 0.15 to 0.27 min. The mice were monitored using the video tracking system during the SD time. Moreover, EEG recording was performed during the 4-h SD paradigm.

### Statistical Analysis

To compare the sleep behavior between the three phenotypes, we used mixed-effects model, instead of two-way repeated measures ANOVA, to account for the missing values (GraphPad Prism, CA, United States). Time series of percent time, number of bouts, and average duration of bouts were created by quantifying and averaging across 2-h time intervals for a 24-h BL pre-CSD, 24-h BL post CSD and 20-h homeostatic sleep responses pre- and post-CSD. To compare between the different time series such as percent time, number of bouts, average duration of bouts of the three vigilance states (wake, NREM, and REM), a mixed-model two-way ANOVA was performed with between-subjects factor “phenotype” (Susceptible vs. Resilient vs. Stress-naïve) and within-subjects factor “time” (12 × 2 h) for BL recordings. When a significant “phenotype” × “time” interaction was observed, a mixed-model two-way ANOVA was performed with between-subjects factor “phenotype” (Susceptible vs. Resilient vs. Stress-naïve) and within-subjects factor “time” (6 × 2 h) to investigate the light and the dark phases separately. For *post hoc* analyses on time series and average data, we used Tukey’s multiple comparisons test (*p* < 0.05). One sample *t*-tests were performed to measure changes of the 12-h average of % time, number of bouts and average bout duration across post- and pre-CSD.

One-way ANOVA was performed to compare the bouts latencies and the average duration of inter-bout interval during the light and the dark phase pre-and post-CSD. Latency is defined as the time until the first bout of the different vigilance states. Then, a mixed-model two-way ANOVA was used to analyze across conditions to determine the between-subjects factor/interphenotype “phenotype” effect (Susceptible vs. Resilient vs. Stress-naïve) and within-subjects factor/intraphenotype effect such as “stress” (pre-CSD vs. post-CSD) or “phase” (Light vs. Dark). *Post hoc* analysis to assess the inter-phenotype difference was Turkey’s multiple comparisons test (*p* < 0.05) and for intra-phenotype difference across conditions or phases was SIDAK’s multiple comparisons test (*p* < 0.05).

To compare between the different power spectra, a mixed-effects model with between-subjects factor “phenotype” and within-subjects factor “frequency” (90 Hz × 0.5 Hz) was performed. Power spectra of NREM bouts were normalized to the pre-stress baseline average value of SWA (0.5–4.5 Hz) during the 12-h light phase. The low frequency range of normalized SWA (0.5–3 Hz) of NREM bouts, following 4-h SD, was quantified and averaged across 2-h time intervals. Two-way repeated measures ANOVA with “phenotype” as between-subjects factor and “time” (4-h × 2-h) as within-subjects factor was performed. Friedman’s Aligned Rank test was used to compare between the average cumulative duration of NREM and REM bouts of the homeostatic sleep response post SD (*p* < 0.05).

To build a predictive model for resilience and susceptibility using pre-stress sleep features, feature engineering using SelectKBest algorithm (*F*-test) and Variance Inflation Factor (VIF) to detect collinearity between features were performed. Logistic regression model was built in Python using the Scikit-learn library. Cross-validation of the predictive model was performed by splitting the data into 75% training set and 25% test set.

## Results

### Pre-exposure to Stress, Resilient Mice Sleep Less Than the Susceptible Mice

#### During the Dark

**FIGURE 1:**
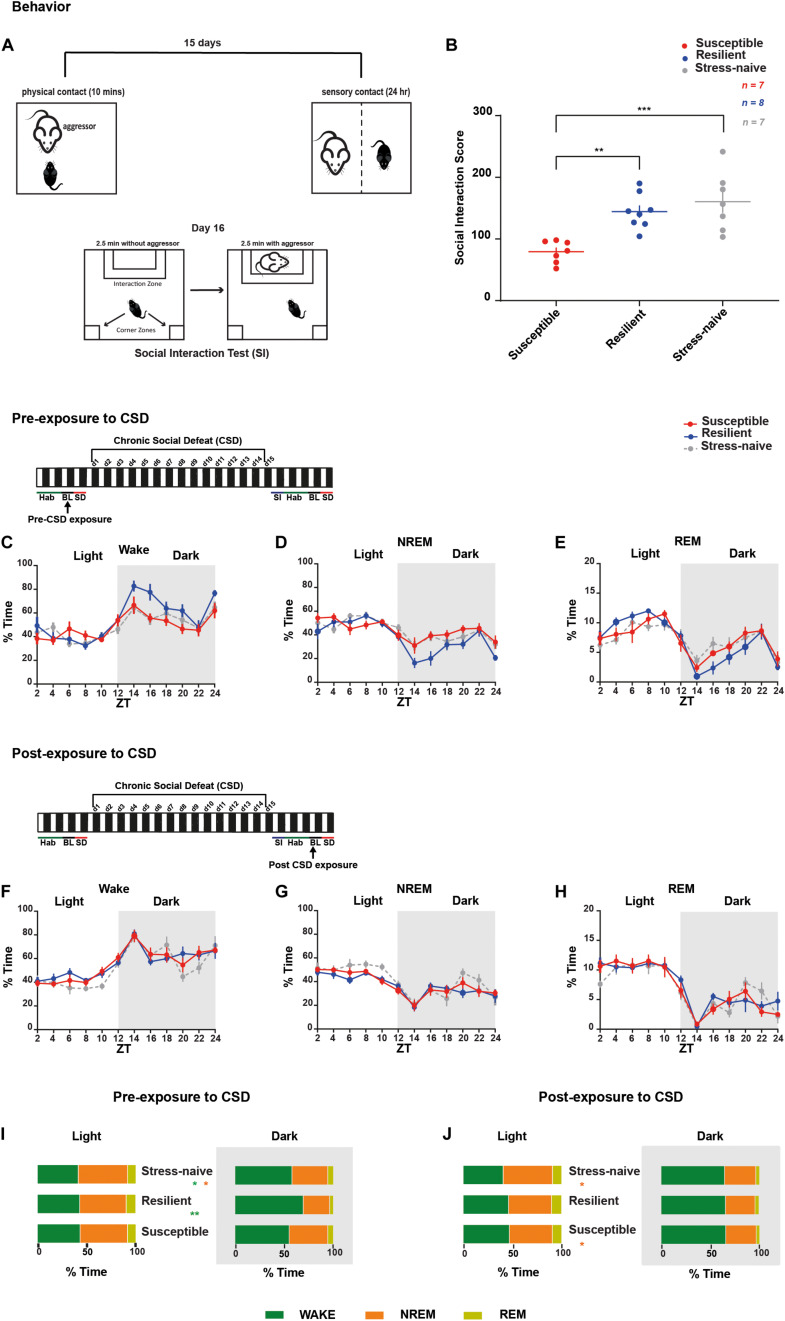
Sleep and wake states were assessed in mice pre- and post-CSD paradigm. Mice resilient to stress spent more time awake in the dark during pre-, but not post-exposure, to CSD stress. **(A,B)** Overview of the chronic social defeat (CSD) paradigm. **(A)** C57BL/6J mice were introduced into the cage of a novel and aggressive CD1 mouse for 10-min (physical aggression), then separated by a clear perforated plexiglass divider for the following 24-h (psychological aggression). Mice were exposed to a novel CD1 every day for 15 days. On day 16, the social interaction test was performed to assess the social avoidance behavior. **(B)** Social interaction score is defined as the percent ratio of the time spent in target-interaction zone to the time spent in no-target interaction zone. Both the stress-naïve and resilient mice exhibit a score >100, while the susceptible mice exhibit a score <100. There was a phenotype effect (*F*_2_,_19_ = 11.62, *p* < 0.001) as both resilient and stress-naïve exhibited a higher score than susceptible mice (*p* < 0.01 and *p* < 0.001, respectively). **(C–E)** *Pre-CSD stress:* There was a “time” effect across all vigilance states (*p* < 0.0001). **(C)** Wake: There was a “time” x “phenotype” interaction effect (*F*_22_,_202_ = 1.736, *p* < 0.05). In the dark, there was a trend of “phenotype” effect (*F*_2_,_18_ = 3.11, *p* = 0.069). **(D)** NREM sleep: There was a “time” x “phenotype” interaction effect (*F*_22_,_202_ = 1.790, *p* < 0.05). In the dark, there was a trend of “phenotype” effect (*F*_2_,_18_ = 3.162, *p* = 0.067). **(F–H)** Post-CSD stress: There was a significant effect of “time” in all three subfigures (*p* < 0.0001). **(I,J)** A summary of the main findings showing: *Pre-CSD stress:* **(I)** There was no difference in time spent in the different vigilance states between the phenotypes in the light. Resilient mice spent more time awake relative to susceptible and stress-naive mice in the dark (*p* < 0.01 and *p* < 0.05, respectively). *Post-CSD stress*: **(J)** Susceptible and Resilient mice spent less time in NREM sleep relative to stress-naïve mice during the light (*p* < 0.05 and *p* < 0.05, respectively). No difference was observed between the phenotypes in the amount of time spent in the different vigilance states during the dark. Values are expressed as percentage of total recording time (mean ± sem) averaged across 2-h **(C–H)** or 12-h intervals **(I,J)**. *n* = 7–8 for each group.

For 15 days, C57BL/6J mice were exposed to daily physical aggression from a novel CD1 aggressor mice for 10 min followed by 24-h psychological aggression since the test mice were kept in the same cage but separated with a perforated plexiglass divider from the aggressor (Figure 1A). CSD-exposure led to the grouping of the mice into susceptible and resilient phenotypes, based on their social avoidance behavior that was assessed on day 16 via the SI test. Stress-exposed mice were classified as resilient if they exhibited a score >100 or as susceptible mice if they exhibit a score <100 (Figure 1B and Supplementary Figure 1B). Prior to CSD, we measured the percent of time all the mice spent in the different vigilance states prior to their exposure to CSD during the light and dark phases (Figures 1C–E,I and Supplementary Figures 2A,B). There was a significant effect of “time” (*p* < 0.0001) for all vigilance states. There was an interaction between “phenotype” × “% time” of wake (*F*_22_,_202_ = 1.74, *p* < 0.05; Figure 1C) and between “phenotype” × “% time” of NREM (*F*_22_,_202_ = 1.79, *p* < 0.05; Figure 1D). Pre-CSD, the resilient mice spent more time awake in the dark (*p* < 0.01; Figure 1I and Supplementary Figure 2B) by spending less time in NREM sleep (*p* < 0.05; Figure 1I and Supplementary Figure 2B) compared to the susceptible mice. Post exposure to CSD, there were no striking differences in the time spent in the vigilance states between susceptible and resilient mice (Figures 1F–H,J and Supplementary Figures 2C,D). However, both susceptible and resilient mice spent less time in NREM sleep compared to stress-naïve mice in the light (*p* < 0.05 and *p* < 0.05, respectively; Figure 1J and Supplementary Figure 2C). In summary, pre-CSD, resilient mice spent more time in the wake state in the dark relative to susceptible and stress-naïve mice (*p* < 0.01 and *p* < 0.05, respectively; Supplementary Figure 2B). The effect of chronic stress-exposure on both resilient and susceptible mice resulted in reduced NREM sleep time in the light, compared to stress-naive mice. In light of our observation that resilient mice spent more time awake in the dark prior to stress, future studies will investigate whether longer wake duration during the dark confers resilience to future stress.

### Susceptible Mice Exhibit Greater Number of Wake and NREM Bouts Pre- and Post-CSD

**FIGURE 2:**
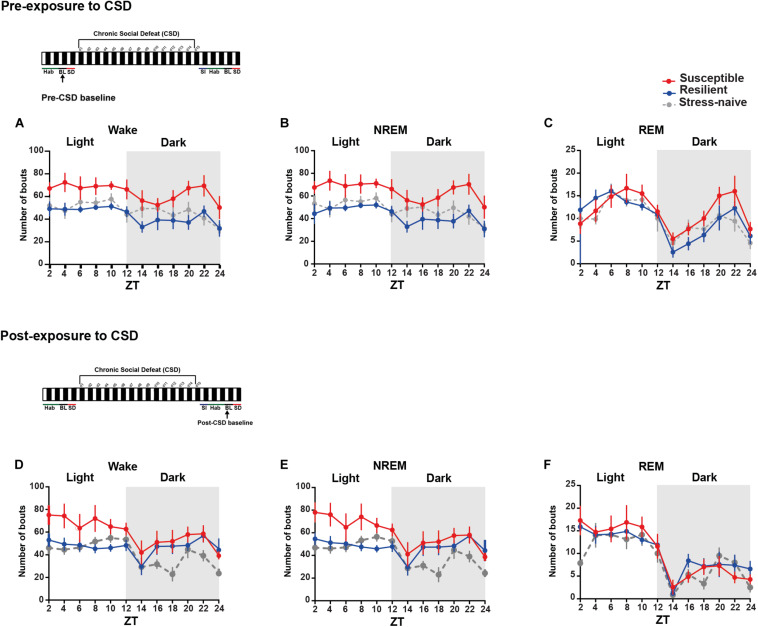
Mice susceptible to stress displayed greater number of wake and NREM bouts pre-and post-CSD stress. **(A–C)** *Pre-CSD stress:* Susceptible mice displayed a higher number of **(A)** wake and **(B)** NREM bouts (*F*_2_,_19_ = 4.314, *p* < 0.05, and *F*_2_,_19_ = 4.456, *p* < 0.05, respectively). **(D–F)** *Post-CSD stress*: **(D)** There was a trend of higher number of wake bouts in susceptible mice relative to resilient and stress-naïve groups (*F*_2_,_19_ = 3.395, *p* = 0.055). **(E)** There was “phenotype” effect in the number of NREM bouts (*F*_2_,_19_ = 4.275, *p* < 0.05). Values are expressed as the number of bouts across 2-h intervals (mean ± sem). *n* = 7–8 for each group.

**FIGURE 3:**
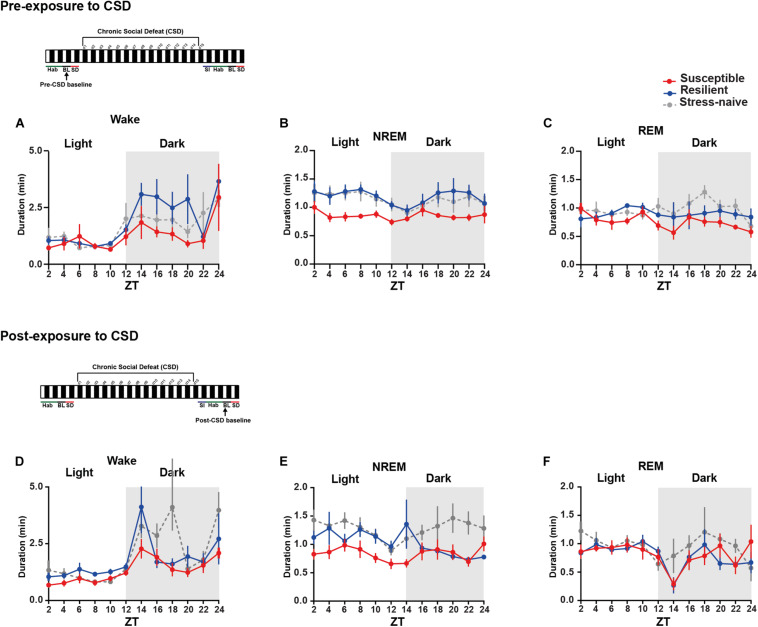
Mice susceptible to stress exhibited shorter average duration of NREM bouts pre-and post-CSD stress in the light. **(A–C)** *Pre-CSD stress:* **(B)** The average duration of NREM bouts was shorter in the susceptible mice relative to resilient and stress-naïve mice (*F*_2_,_19_ = 3.278, *p* = 0.06). **(D–F)** *Post-CSD stress:* **(E)** The average duration of NREM bouts of susceptible mice was shorter than the average NREM bout duration of resilient and stress-naïve mice in the light (*F*_2_,_19_ = 5.05, *p* < 0.05). In the dark, an effect of “phenotype” (*F*_2_,_15_ = 4.082, *p* < 0.05), when excluding the first 2-h, was due to the fact that the average duration of NREM bouts of susceptible mice was shorter relative to stress-naïve mice only. Values are expressed as average bout duration (mean ± sem) across 2-h intervals. *n* = 7–8 for each group.

We were then interested in investigating the detailed dynamics of the sleep architecture by calculating the number and average duration of bouts of the vigilance states during the light and the dark pre- and post- exposure to stress (Figures 2, 3A–F). Pre-CSD, mice susceptible to stress showed significantly higher number of wake bouts (*F*_2_,_19_ = 4.314, *p* < 0.05; Figure 2A and textitlight: p < 0.05, *dark: p* < 0.001; Supplementary Figures 2E–F) and NREM bouts (*F*_2_,_19_ = 4.456, *p* < 0.05; Figure 2B and textitlight: p < 0.01, *dark: p* < 0.001; Supplementary Figures 2E,F) compared to resilient mice, which suggests increased NREM fragmentation. Post-CSD, there was an interaction between “time” × “phenotype” for wake bouts (*F*_22_,_185_ = 1.869, *p* < 0.05; Figure 2D) and significant interaction between “time” × “phenotype” for NREM bouts (*F*_22_,_185_ = 3.56, *p* < 0.05; Figure 2E). During the light phase, susceptible mice showed higher number of wake bouts (*F*_2_,_19_ = 3.395, *p* = 0.055; Figure 2D and textitp < 0.01, *p* < 0.05, respectively; Supplementary Figure 2G) and NREM bouts (*F*_2_,_19_ = 4.275, *p* < 0.05; Figure 2E and textitp < 0.01, *p* < 0.05, respectively; Supplementary Figure 2G) relative to resilient and stress-naïve mice. During the dark, there was a significant effect of “time” in wake and NREM bouts (*p* < 0.0001for both; Figures 2D–E, Supplementary Figure 2H), while there was a trend of greater number of wake and NREM bouts in susceptible compared to stress-naïve mice (*p* = 0.056 and *p* = 0.059; Supplementary Figure 2H).

In short, the increase in the number of wake and NREM bouts, pre-CSD, is correlated with vulnerability to stress.

### Susceptible Mice Exhibit Shorter Average NREM Bout Duration Pre- and Post-CSD

We then measured the average duration of the bouts of the different vigilance states among the three phenotypes during the light and the dark pre- and post-CSD (Figures 3A–F and Supplementary Figures 2I,L). Pre-CSD, there was a significant effect of “time” for wake and NREM bouts (*p* < 0.0001 and *p* < 0.05, respectively; Figures 3A,B). Mice susceptible to stress exhibited shorter average duration of NREM bouts than resilient and stress-naïve mice (*F*_2_,_19_ = 3.278, *p* = 0.06; Figure 3B and textitlight: *p* < 0.01, *p* < 0.01, respectively; Supplementary Figure 2I). Similarly, post-CSD, mice susceptible to stress exhibited shorter average duration of NREM bouts than stress-naïve mice in the light (*F*_2_,_19_ = 5.05, *p* = 0.056; Figure 3E and textitp < 0.05; Supplementary Figure 2K). There was a significant interaction between “time” × “phenotype” (*F*_22_,_185_ = 1.778, *p* < 0.05; Figure 3E). In fact, post-CSD, during the dark, excluding the first 2-h, both susceptible and resilient mice exhibited significantly shorter average duration of NREM bouts compared to stress-naïve mice as there was a significant “phenotype” effect (*F*_2_,_15_ = 4.082, *p* < 0.05; Figure 3E and Supplementary Figure 2L). The first 2 h, (ZT = 12–14), were excluded as resilient mice at this point exhibited NREM average bout duration comparable to stress-naïve mice.

In summary, the shorter average duration of NREM bouts, pre-CSD, is associated with vulnerability to stress. Additionally, observation that the average duration of NREM bouts, post-CSD, during the dark is shorter in both susceptible and resilient mice is indicative of a common response to stress in both phenotypes. Moreover, by comparing sleep and wake states pre- and post-CSD, we observed the following changes: resilient mice spent less time in NREM sleep during the light, while susceptible mice spent more time in Wake and less time in NREM and REM sleep during the dark, post-CSD relative to pre-CSD. Such change was accompanied by a decrease in the number of REM bouts of susceptible mice post-CSD relative to pre-CSD during the dark (*p* < 0.05 for all tests; Supplementary Figures 3, 4).

### Susceptible Mice Exhibit Greater Switching Between Wake and NREM Pre- and Post-CSD

**FIGURE 4:**
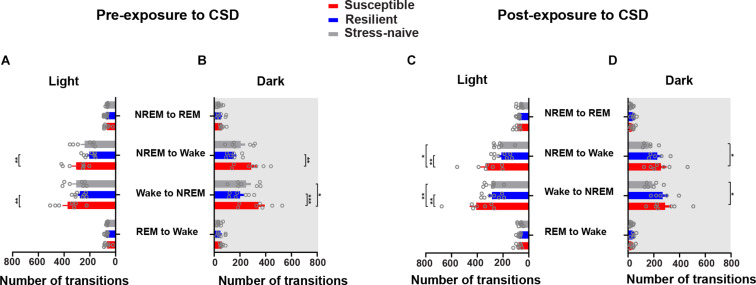
Susceptible mice displayed a higher number of state transitions between NREM and wake during the light and dark phases pre- and post-exposure to CSD. *Pre-CSD*: **(A)** The numbers of transitions from wake to NREM and from NREM to wake were higher in susceptible mice relative to resilient mice (*p* < 0.01 and *p* < 0.01, respectively) during the light. **(B)** In the dark, the numbers of transitions from wake to NREM and from NREM to wake were higher in susceptible mice relative to resilient (*p* < 0.0001 and *p* < 0.001, respectively) and stress-naïve mice (*p* < 0.05 and *p* = 0.066, respectively). *Post-CSD:* **(C)** In the light, the numbers of transitions from wake to NREM and from NREM to wake were higher in susceptible mice compared to resilient (*p* < 0.01 and *p* < 0.01, respectively) and stress-naïve mice (*p* < 0.01 and *p* < 0.05, respectively). **(D)** In the dark, the numbers of transitions from the wake to NREM and from NREM to wake were higher in susceptible mice compared to stress-naïve mice (*p* < 0.05 and *p* < 0.05, respectively). Values are expressed as number of transitions across 12-h intervals (mean ± sem). *n* = 7–8 for each group. *Post hoc* analyses using Tukey’s multiple comparisons were performed.

The similar increases in the number of bouts of wake and NREM in the susceptible mice suggests increased switching between these two states. Therefore, we computed the number of transitions between the vigilance states such as the number of transitions from REM to wake, wake to NREM, NREM to wake and NREM to REM (Figures 4A–D). The numbers of transitions from wake to NREM and from NREM to wake were significantly greater during the light in susceptible mice pre-CSD relative to resilient mice (*p* < 0.01 and *p* < 0.01, respectively; Figure 4A). During the dark, the numbers of transitions from wake to NREM and from NREM to wake continue to be greater in susceptible mice pre-CSD relative to resilient and stress-naïve mice (resilient: *p* < 0.001 and *p* < 0.01; stress-naïve: *p* < 0.05 and *p* = 0.066, respectively; Figure 4B). Post-CSD, during the light, the numbers of transitions from wake to NREM and from NREM to wake were greater in susceptible mice compared to resilient and stress-naïve mice (resilient: *p* < 0.01 and *p* < 0.01, respectively; stress-naïve: *p* < 0.01 and *p* < 0.05, respectively; Figure 4C). During the dark, susceptible mice displayed greater number of wake to NREM and NREM to wake transitions relative to stress-naïve mice (*p* < 0.05 and *p* < 0.05, respectively; Figure 4D).

Our findings show that susceptible mice exhibit greater NREM sleep fragmentation prior to stress because of: (i) the similar increases in the number of wake and NREM bouts, (ii) the decrease in the average duration of NREM bouts and (iii) the increased switching between NREM and wake bouts. This finding indicates that fragmented NREM sleep may be a predictor of vulnerability to future stress. Moreover, the increase in NREM fragmentation persisted post exposure to stress in susceptible mice. Interestingly, the mice resilient to stress displayed higher fragmentation of NREM, post exposure to stress, but only in the dark. We also assessed the latencies and average duration of interbout interval pre-and post-CSD stress (Supplementary Figures 5, 6, respectively). No difference was observed in the latencies of the vigilance states of all phenotypes pre-and post-CSD (Supplementary Figure 5 and Supplementary Table 1). However, pre-CDS, susceptible mice displayed shorter average duration of inter-wake interval during the light relative to resilient and stress-naïve mice (*p* < 0.05 and *p* < 0.05, respectively; Supplementary Figure 6AI). Furthermore, post-CSD, susceptible mice displayed shorter average duration of inter-wake interval during the dark compared to stress-naïve mice (*p* < 0.05; Supplementary Figure 6DII), and significantly shorter average duration of inter-NREM interval during the light relative to resilient mice (*p* < 0.05; Supplementary Figure 6EI). We further assessed the quality of sleep by analyzing the delta power of NREM episodes pre-and post-CSD (Supplementary Figure 7). There was no difference in the delta power in NREM either between the phenotypes or across pre- and post-CSD (Supplementary Figure 7A). Furthermore, there was no significant change detected in beta and slow gamma power in NREM sleep, which are high frequency EEG bands associated with increased sleep fragmentation and insomnia (Perlis et al., 2001a, b; Riedner et al., 2016; Henderson et al., 2017), from pre- to post-CSD and between the phenotypes (Supplementary Figure 7B). Similarly, in REM sleep episodes, theta power was comparable between the phenotypes and across pre- and post-CSD (Supplementary Figure 7C). Additionally, no significant change in beta or slow gamma power in REM sleep across pre- and post-CSD was observed between the phenotypes (Supplementary Figure 7D).

It is worth mentioning that by combining the features of all vigilance states (percent time, number of bouts, average bout duration) of both susceptible and resilient mice, pre-CSD, into one category labeled stress-exposed mice, they became comparable to the features of the stress-naïve mice. This finding suggests that the sleep wake profile of stress-naïve mice likely consist of a combination of sleep wake profiles from both categories (Supplementary Figures 8A–I, 9A–D).

### Homeostatic Sleep Response Pre-exposure to CSD

**FIGURE 5:**
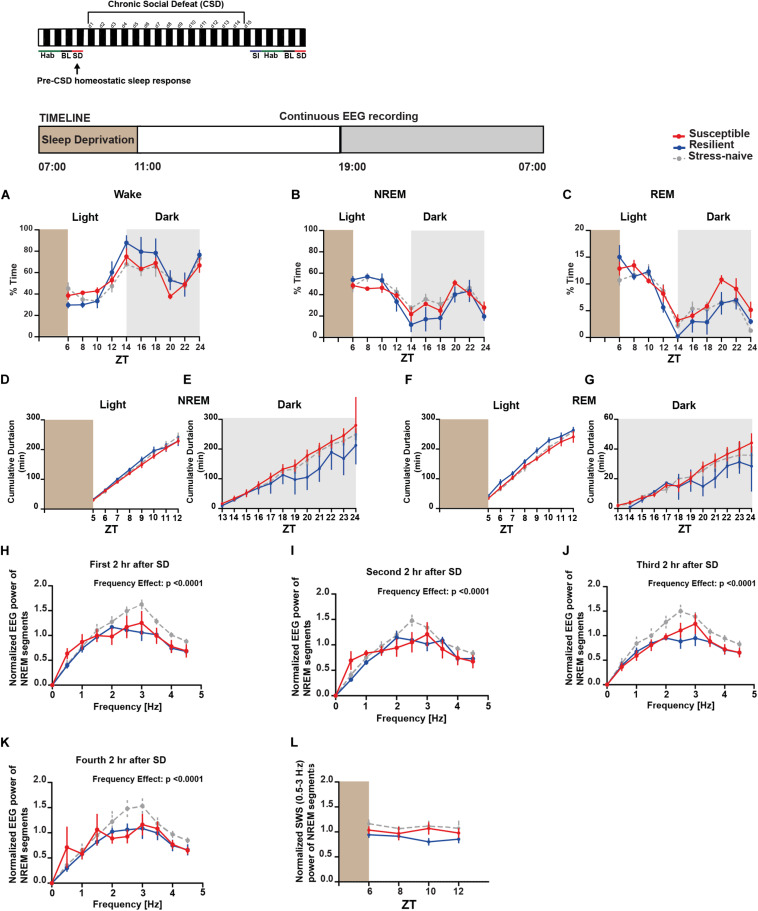
*Pre-CSD stress:* homeostatic sleep response was comparable between the three phenotypes. **(A–C)** There was no difference in the time spent in the different vigilance states among the three phenotypes post SD. **(D)** Resilient mice spent more time in NREM as the time progressed during the light (*p* < 0.05), and **(E)** displayed lower average cumulative duration of NREM during the dark as they spent more time awake (*p* < 0.05) relative to susceptible mice. **(F)** Additionally, resilient mice exhibited greater average cumulative duration of REM during the light relative to stress-naïve and susceptible mice (*p* < 0.05 and *p* < 0.05, respectively), and **(G)** exhibited lower average cumulative duration of REM during the dark as they spent more time awake relative to susceptible (*p* < 0.05). **(H–K)** Power spectral analysis of EEG recordings of NREM state 2, 4, 6, and 8-h after SD. Power spectra are normalized to the pre-stress baseline average value of SWA (0.5–4.5 Hz) during the 12-h light phase. **(L)** The low frequency range of normalized SWA (0.5–3 Hz) of NREM bouts, following 4-h SD, was quantified and averaged across 2-h time intervals. There was no difference in normalized SWA (0.5–3 Hz) of NREM bouts between the 3 phenotypes following SD. Values are expressed as percentage of total recording time **(A–C)** or normalized power **(H–L)** (mean ± sem). *n* = 4–6 for each group.

Moreover, we were interested in assessing the difference in the homeostatic sleep response pre-and post-CSD in both the susceptible and resilient mice relative to stress-naïve mice. We investigated the homeostatic sleep response in mice after 4-h SD applied at the start of the light phase pre-CSD (Supplementary Figure 10A). There was no difference in the amount of time the mice from the three groups spent in the different vigilance states (Figures 5A–C). We computed the cumulative duration of NREM and REM post SD (Figure 5D-G). Resilient mice had higher average cumulative duration of NREM post SD during the light relative to susceptible mice (*p* < 0.05; Figure 5D), while they displayed lower average cumulative duration of NREM during the dark as they spent more time awake relative to susceptible mice (*p* < 0.05; Figure 5E). Resilient mice exhibited greater average cumulative duration of REM relative to susceptible and stress-naïve mice during the light (*p* < 0.05 and *p* < 0.05, respectively; Figure 5F), while they exhibited lower average cumulative duration of REM during the dark as they spent more time awake relative to susceptible mice (*p* < 0.05; Figure 5G). Since enhanced slow wave amplitude (SWA; 0.5–4.5 Hz) is observed following SD and as it is a marker for sleep homeostasis (Daan et al., 1984), we computed the power spectra of NREM, that encompasses predominantly the frequencies of SWA, 2, 4, 6, and 8-h after SD, averaged across 2-h intervals. There was a significant effect of within-subjects factor “frequency” on all panels (*p* < 0.0001; Figures 5H–K). We assessed the homeostatic response by quantifying the low frequency range of normalized SWA (0.5–3 Hz) across 2-h intervals, after SD, for the remaining duration of the light phase. There was no significant difference in the SWA among the three phenotypes (Figure 5L).

### Aberrant Homeostatic Sleep Response in Susceptible and Resilient Mice Post-exposure to CSD

**FIGURE 6:**
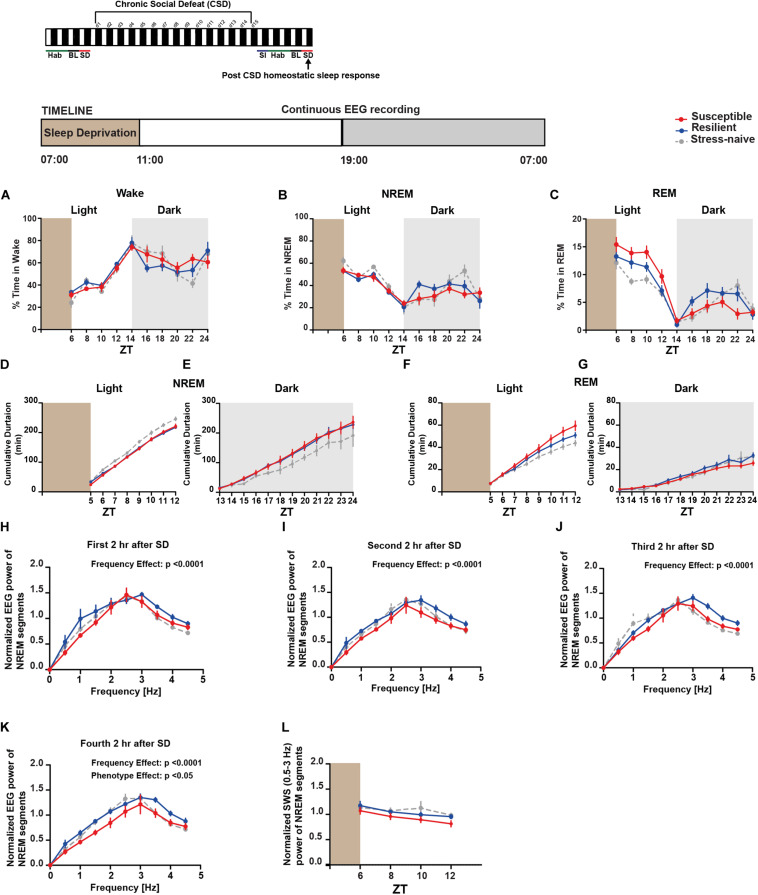
*Post-CSD Stress:* Aberrant homeostatic sleep response of stress-exposed mice. **(A–C)** During the light, there was “phenotype” effect in the REM homeostatic sleep response as the susceptible mice spent more time in REM sleep than the resilient and the stress-naïve mice (*F*_2_,_18_ = 6.098, *p* < 0.01; **C**). **(D)** Stress-naive mice exhibited greater average cumulative duration of NREM relative to susceptible and resilient mice (*p* < 0.05 for both comparisons). **(E)** Susceptible and resilient mice displayed greater average cumulative duration of NREM during the dark relative to stress-naive (*p* < 0.05 for both comparisons). **(F)** Susceptible mice displayed the greatest average cumulative duration of REM, followed by resilient and stress-naïve mice during the light (*p* < 0.05 for all comparisons). **(G)** Resilient mice exhibited greater average cumulative duration of REM during the dark relative to susceptible mice (*p* < 0.05). **(H–K)** Normalized power spectral analysis of EEG recordings 2, 4, 6, and 8-h after SD. There was a significant effect of within-subjects factor “frequency” on all 4 subfigures (*p* < 0.0001). **(K)** There was a “phenotype” effect in the interval (6–8-h) post SD (*F*_2_,_16_ = 3.697, *p* < 0.05) as susceptible mice displayed lower spectral power in the band (0.5–4.5 Hz) compared to resilient and stress-naïve mice. **(L)** Repeated measures two-way ANOVA of normalized SWA (0.5–3 Hz) of NREM bouts showed a significant effect of time (*F*_1_._732_,_29_._45_ = 4.672, *p* < 0.05). Data are averaged across 2-h intervals. Values are expressed as percentage of total recording time **(A–C)** or normalized power **(H–L)** (mean ± sem). *n* = 6–8 for each group.

Next, we investigated the homeostatic sleep response in mice after 4-h SD applied at the start of the light post-CSD (Supplementary Figure 10B). There was a significant interaction between “phenotype” × “time” for NREM (*F*_18_,_156_ = 1.81, *p* < 0.05; Figure 6B) and REM (*F*_18_,_156_ = 3.087, *p* < 0.0001; Figure 6C). The susceptible mice spent more time in REM sleep, post SD, relative to the resilient and stress-naïve mice during the light phase (*F*_2_,_18_ = 6.098, *p* < 0.01; Figure 6C). We next computed the cumulative duration of NREM and REM post SD (Figures 6D–G). Stress-naive mice exhibited greater average cumulative duration of NREM relative to susceptible and resilient mice during the light (*p* < 0.05 and *p* < 0.05, respectively; Figure 6D). Both susceptible and resilient mice displayed greater average cumulative duration of NREM during the dark phase relative to stress-naive mice (*p* < 0.05 and *p* < 0.05, respectively; Figure 6E). Susceptible mice displayed the greatest average cumulative duration of REM, followed by resilient and stress-naïve mice during the light phase post SD (*p* < 0.05 for all comparisons; Figure 6F). Resilient mice exhibited greater average cumulative duration of REM during the dark relative to susceptible mice (*p* < 0.05; Figure 6G). We then computed the power spectra of the NREM sleep 2, 4, 6, and 8-h after SD, averaged across 2-h intervals (Figures 6H–K). There was a significant effect of within-subjects factor “frequency” on all four subfigures (*p* < 0.0001; Figures 6H–K) and a significant effect of between-subjects factor of “phenotype” (*p* < 0.05; Figure 6K). We assessed the homeostatic response by quantifying the low frequency range of normalized SWA (0.5–3 Hz) across 2-h intervals, after SD, for the remaining duration of the light phase. There was a significant effect of time (*F*_1_._732_,_29_._45_ = 4.672, *p* < 0.05; Figure 6L). In summary, the homeostatic response was different between the three phenotypes, as SWA was lower in susceptible mice compared to resilient and stress-naïve mice.

### Predicting Susceptibility and Resilience to Future Stress Using Pre-CSD Sleep Features

**FIGURE 7:**
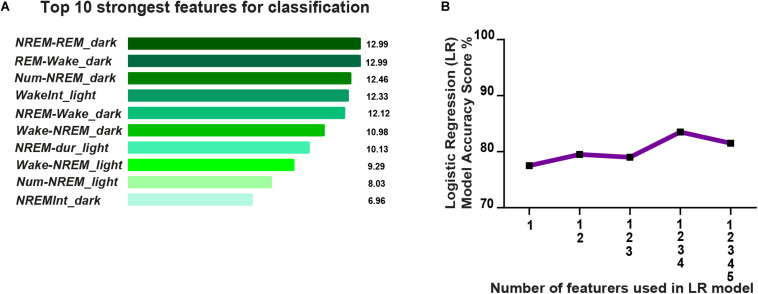
Prediction of resilience versus susceptibility to future stress via logistic regression model using EEG sleep features extracted pre-CSD stress (averaged across 12-h intervals: Light and Dark). **(A)** Top ten pre-CSD stress sleep features, that have the strongest relationship with the prediction output of susceptibility to stress, were ranked using the *F*-test via SelectKBest algorithm. The features are as follows: 1. NREM_REM_dark: number of NREM to REM transitions in the dark, 2. REM_Wake_dark: number of REM to wake transitions in the dark, 3. Num-NREM_dark: number of NREM bouts in the dark, 4. WakeInt_L: average duration of inter-wake interval in the light, 5. NREM-Wake_D: number of transitions of NREM to Wake in the dark, 6. Wake-NREM_D: number of transitions of wake to NREM bouts in the dark, 7. NREM-dur_L: average duration of NREM bouts in the light, 8. Wake-NREM_L: number of transitions of Wake to NREM in the light, 9. Num-NREM_L: number of NREM bouts in the light, and 10. NREM-Int_D: average duration of inter-NREM interval in the dark. **(B)** Accuracy of the logistic regression model improves as more top features are integrated and reaches performance of 83.5% with the top 4 features, then declines due to potential overfitting.

Next we investigated the possibility of predicting resilience versus susceptibility to future stress using previously assessed pre-CSD sleep features. Therefore, we built a simple classifier, using a linear logistic regression model, and trained it on pre-CSD sleep features from a subset of susceptible and resilient mice, that were both CSD-stress naïve at this point. We then tested the classifier on a test set of pre-CSD sleep features from the remaining susceptible and resilient mice and predicted the phenotype for cross-validation purpose. To build the classifier, we gathered 24 pre-CSD sleep features, where some exhibited a linear, albeit weak, relationship with SI (Supplementary Figure 11 and Supplementary Table 7). We then used feature engineering to select the top ten pre-CSD stress sleep features (Supplementary Table 7) that had the strongest relationship with the prediction output of susceptibility to stress using *F*-test, by ranking them based on the ANOVA *F*-values (Figure 7A). In order to minimize the multicollinearity between the features, we used VIF, which is used to determine the strength of correlation between independent variables. We eliminated features with VIF > 35, which yielded the following **5 top** features with reduced multicollinearity: 1. Number of transitions from REM to Wake in the dark 2. Number of transitions of wake to NREM in the dark 3. Average NREM bout duration in the light 4. Number of transitions of wake to NREM in the light 5. Average duration of inter-NREM interval in the dark. We gradually incorporated these features into the logistic regression model and computed the model accuracy, via cross-validation, at each addition (Figure 7B). The accuracy reached 83.5% with the top 4 features, then dropped due to potential overfitting by adding the additional feature. Principal component analysis showed a separation of the susceptible and resilient clusters using 2, 3, 4, and 5 top features (Supplementary Figures 12A–D).

## Discussion

The present study assessed the potential role of sleep in the etiopathogenesis of depression-like behavior by analyzing sleep-wake patterns prior and post exposure to chronic social stress. Mice susceptible to future stress exhibited greater NREM sleep fragmentation relative to resilient mice, which demonstrates that abnormal sleep is a putative marker of stress-vulnerability. Clinical correlational studies investigating the link between sleep abnormalities and mood disorders have led to the growing body of evidence of the causal role of sleep in regulating emotional brain function (Walker and van der Helm, 2009). Since comorbidity between sleep alterations and mood disorders is common, sleep disruption is a core symptom for the diagnosis of depression. Typical sleep alterations in these patients include reduced NREM sleep, shortened REM sleep latency, increased REM density and duration and compromised sleep continuity (Riemann et al., 2020). The relationship between sleep and depression is bi-directional. Indeed, it is hypothesized that some antidepressants are effective in part due to their direct action on improving sleep quality in terms of latency to sleep, sleep consolidation and NREM stability. Such evidence suggests a mechanistic link at the molecular and neurophysiological level between sleep and depression (Wulff et al., 2010). Furthermore, accumulating evidence implicates that sleep disturbances preceding exposure to stress leads to poor psychiatric outcomes (Parrino and Vaudano, 2018). Specifically clinical data provide strong evidence of the bi-directional relationship between insomnia and depression by implicating insomnia or disturbances in sleep continuity as an independent predictor for depression (Batterham et al., 2012; Kalmbach et al., 2018; Riemann et al., 2020).

Despite substantial clinical evidence, there has been limited basic research into investigating pre-existing sleep abnormalities as a predictor for vulnerability to depressive disorders. To our knowledge, there has been only one direct investigation that demonstrated a correlation between prior sleep disturbances and mood disorders (Polta et al., 2013). The study only showed the correlation between the number of REM bouts pre-stress (baseline condition) and hyperarousal following exposure to a fear conditioning paradigm. Therefore, we aimed to address the lack of available animal models for investigating the association between pre-existing sleep abnormalities and vulnerability to chronic social stress. We assessed the sleep of mice pre-and post-exposure to CSD, which is a widely validated preclinical model of major depression disorder (MDD) (Krishnan et al., 2007; Golden et al., 2011; Chaudhury et al., 2013).

Pre-CSD, all three groups of mice spent the same amount of time in the different vigilance states during the light phase, while in the dark phase the stress-resilient mice slept less, relative to the stress-susceptible mice. Closer examination of the sleep architecture such as bout dynamics **prior** to stress revealed interesting differences between the stress-vulnerable and stress-resilient mice that could be potential markers of stress vulnerability. Specifically, pre-CSD, susceptible mice exhibited higher numbers of wake and NREM bouts and increased switching between NREM and wake states leading to greater NREM sleep fragmentation during both the light and dark phases. This was confirmed by shorter average duration of NREM bouts in susceptible mice pre-CSD, relative to the resilient and stress-naïve mice. Our finding provides strong evidence of an association between poor sleep continuity and vulnerability to stress. The poor consolidation of NREM sleep in the susceptible mice might compromise sleep homeostatic processes that regulate synaptic plasticity via downscaling of synaptic strength (Bryant et al., 2010; Parrino and Vaudano, 2018; Radwan et al., 2019). Moreover, our results are consistent with previous reports of increased risk of depression among patients with insomnia (Ford and Kamerow, 1989; Breslau et al., 1996; Chang et al., 1997; Perlis et al., 1997; Franzen and Buysse, 2008; Batterham et al., 2012, 2017; Fernandez-Mendoza et al., 2016; Li et al., 2016; Goldschmied et al., 2019). The leak K^+^ channels (*Kcnk9*) play a role in generating the down cortical state and the slow wave sleep (SWS) firing pattern (Yoshida et al., 2018). Thus, a potential mechanism underlying the hyper-fragmentation of NREM sleep that we observed might be aberrant leak *Kcnk9* channels in the susceptible mice and it will be worthwhile to explore this hypothesis in the future. It is worth noting that, pre-CSD, we found that sleep, in terms of percent time, number of bouts and average bout duration, was comparable between the stress-exposed (susceptible + resilient) mice and stress-naïve mice. This suggests that the sleep and wake profile of stress-naïve group is made up of a combination of the sleep and wake profiles from both categories of stress-vulnerable and resilient mice. Therefore, some of the sleep features of the stress-naïve mice such as% Time, number of bouts and average bout duration might be a weighted “average” of the features from both susceptible and resilient mice. Further investigation is required to address such a possibility by applying non-supervised learning techniques on sleep-wake features from a greater number of stress-naïve mice than our current study, prior to stress, to investigate: (i) whether there is an inherent clustering into two phenotypes, (ii) the inherent ratio or representation of both vulnerable and resilient group features in the stress-naïve group, and (iii) whether factors such as differences in social hierarchy or early-life maternal stress lead to the generation of the sleep disturbances characterizing the stress-vulnerable phenotype.

Post-CSD, in the light phase, both resilient and susceptible-mice exhibited lower percentage of time in NREM sleep compared to stress-naïve mice. Moreover, the susceptible mice continued to maintain hyper-fragmented NREM sleep during both the light and dark phases, which is reminiscent of the high co-occurrence of insomnia (fragmented sleep) observed in patients with depression (Manber et al., 2016; Gebara et al., 2018). Previous studies have reported other features of insomnia such as augmented high-frequency EEG activity in susceptible mice post CSD (Henderson et al., 2017), which we could not replicate in our dataset. Conversely, resilient mice, during the dark, but not the light phase, exhibited greater fragmented NREM bouts similar to the susceptible mice. The resilient mice are hypothesized to undergo greater changes in homeostatic plasticity that enable them to “buffer” against stress-induced changes observed in susceptible-mice (Krishnan et al., 2007; Chaudhury et al., 2013; Friedman et al., 2014). Our observation that NREM sleep, was negatively affected by stress exposure in the resilient mice, only during the dark, implies that sleep during the light, but not the dark phase, could be potentially involved with homeostatic processes such as synaptic downscaling (Vyazovskiy et al., 2008; Tononi and Cirelli, 2012, 2019; Kuhn et al., 2016) that confers resilience to stress. Moreover, these observations further highlight the interplay between sleep and the circadian system or sleep and light or all three in regulating brain homeostatic processes (Deboer, 2018). Finally, increased sleep pressure, due to prolonged time in the wake state, leads to higher synchronization during NREM sleep (Aeschbach and Borbely, 1993) that could be assessed by measuring SWA (Daan et al., 1984). We observed that such response was impaired in mice following stress-exposure. Moreover, our observation of aberrant SWA response following SD in the susceptible mice, post exposure to stress, agrees with previous studies (Olini et al., 2017; Fujii et al., 2019).

The effect of CSD stress on subsequent sleep has been investigated in prior studies. In one study, there was an increase in the number of REM bouts 5-days post CSD (Wells et al., 2017). In another study, there was a difference in the homeostatic response between stress-exposed and stress-naïve mice following SD (Olini et al., 2017). The differences between the behavior paradigms, the experimental/recording timeline, and context could account for differences in the results in the sleep/wake architecture observed between these studies (Fujii et al., 2019). Additionally, the variability in the aggression level of the CD1 mice (duration, frequency of attacks), the time of the physical contact, the difference in the early life stresses and social ranks among C57BL/6J might also account for some of the differences (Fujii et al., 2019). The criteria used to categorize the susceptible and the resilient phenotypes vary between labs which is another potential cause for differences in findings between the various studies (Henderson et al., 2017; Wells et al., 2017). Furthermore, several of the studies assessed the effect of acute, not chronic, social defeat stress on sleep (Wells et al., 2017; Fujii et al., 2019; Feng et al., 2020) and many focused on REM sleep abnormalities in association with the depressive-like phenotype, despite observations that the diagnostic value of REM sleep abnormalities as a marker of depression has been shown to be confounded with age and gender effects (Thase et al., 1998). In our study we also observed REM sleep abnormalities in terms of higher number of REM sleep bouts in the dark, pre-CSD, in mice that become susceptible to stress (*p* = 0.056) and augmented REM sleep rebound, following SD, in the susceptible mice, post-CSD. However, the main contribution of our study is filling the gap in knowledge about the potential role of pre-existing sleep abnormalities in predicting vulnerability to stress. The increased fragmentation of NREM sleep that we observed in susceptible mice, ***prior*** to CSD exposure, is reminiscent of the role of insomnia and poor sleep continuity in increasing the risk for depression in humans. It is worth noting that there is a paradigm shift in research toward understanding the functional role of insomnia, instead of REM sleep aberrations, as a critical feature of depression (Riemann et al., 2020).

We would like to emphasize that the potential EEG sleep signature for vulnerability observed in our study is **specific** to the CSD stress. We used the EEG sleep features prior to CSD stress exposure to predict susceptibility to stress using a logistic regression model with a high accuracy. It will be interesting in the future to test whether NREM sleep fragmentation converges across different independent models of stress as a biomarker of vulnerability. It is possible that the signature for vulnerability detected in mice that become susceptible to future stress, using sleep EEG in our case or neural spatiotemporal network dynamics in other studies (Hultman et al., 2018; Muir et al., 2018), might be due to other prior stressors such as early life stressors, social hierarchy and other pre-existing factors (Hultman et al., 2018). Indeed, in a study investigating the relationship between sleep and social hierarchy, dominant mice, which have been shown in a previous study to be susceptible to social stress, exhibited fragmented sleep (Larrieu et al., 2017; Karamihalev et al., 2019). In closing, NREM sleep fragmentation in our study is correlated with vulnerability to stress, however, we cannot claim it is a signature of susceptibility that underlies the pathological behavioral state. Further investigation is required to address such distinction.

It would be interesting to extend our study to female mice. It is well established that menstrual hormonal changes influence both sleep duration and sleep quality (Pengo et al., 2018). Additionally, young women have a 28% higher risk of insomnia than their male counterparts (Zhang and Wing, 2006). Moreover, women have a higher risk of depression and anxiety disorders than men (Kornstein et al., 2000; McLean et al., 2011). Thus, it is imperative to investigate whether the increased risk of sleep disturbances is linked to the higher risk of depression in female mice using the repeated social defeat stress model (Takahashi et al., 2017).

Our study lays the foundation for further research into elucidating the role of sleep in the etiopathogenesis of depression. Previous studies reported the effective use of treating sleep abnormalities such as insomnia to alleviate depression (Wulff et al., 2010; Christensen et al., 2016). Our findings further suggest that targeting sleep abnormalities might effectively reduce the risk for depression as demonstrated in previous work (Batterham et al., 2017). Moreover, we highlight the potential validity of using sleep EEG as a biomarker to identify the populations at risk of depression (Rao et al., 2009; Steiger and Pawlowski, 2019). Finally, our study is among the many aiming to provide insights on how sleep might signal emotional pathology, but the first to provide an animal model to investigate the relationship between poor sleep continuity and vulnerability to chronic stress. Having an animal model of sleep signature of vulnerability to stress opens up avenues for many possible future studies aiming to elucidate the underlying molecular processes and neural circuitry that lead to mood disorders.

## Data Availability Statement

The original contributions presented in the study are included in the article/Supplementary Material, further inquiries can be directed to the corresponding author/s.

## Ethics Statement

The animal study was reviewed and approved by the National Institute of Health Guide for Care and Use of Laboratory Animals (IACUC Protocol: 150005A2).

## Author Contributions

BR and DC designed the experiments and wrote the manuscript. BR and GJ collected the data. BR analyzed the data. All authors contributed to the article and approved the submitted version.

## Conflict of Interest

The authors declare that the research was conducted in the absence of any commercial or financial relationships that could be construed as a potential conflict of interest.
