# Supplementary material for: Abnormal Sleep Signals Vulnerability to Chronic Social Defeat Stress
Source: Front Neurosci. 2021 Jan 12;14:610655. doi: 10.3389/fnins.2020.610655 (PMC7835126; doi:10.3389/fnins.2020.610655)
Supplement: Supplementary file 1 [file Data_Sheet_1.pdf]

Supplementary Material

1.1 Supplementary Figures

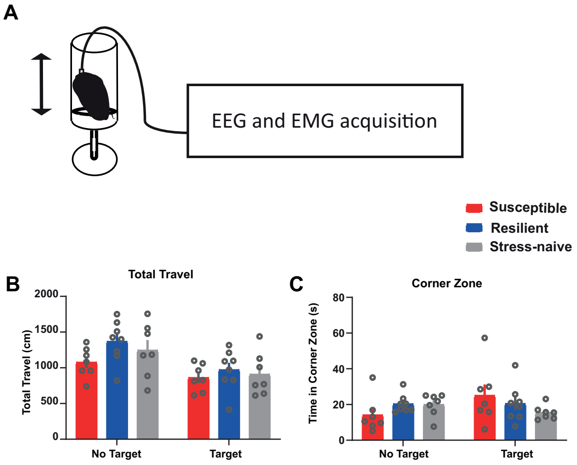

Supplementary Figure 1.

(A) Schematic representation of the cylindrical platform (ViewPoint, Lyon, France) used for sleep recording and deprivation. **(B)** There is no statistical difference in total travel distance between the 3 groups of mice in the absence or presence of a social target (no target: $F_{2,19} = 1.751$, p=0.200; target: $F_{2,19} = 0.330$, p=0.732). **(C)** There is no statistical difference in the time spent in the corner zones between the 3 groups of mice in the absence or presence of a social target (no target: $F_{2,19} = 1.62$, p=0.224; target: $F_{2,19} = 1.242$, p=0.311). Values are expressed as mean ± sem. *n*= 7-8 for each group. All error bars indicate ± s.e.m.

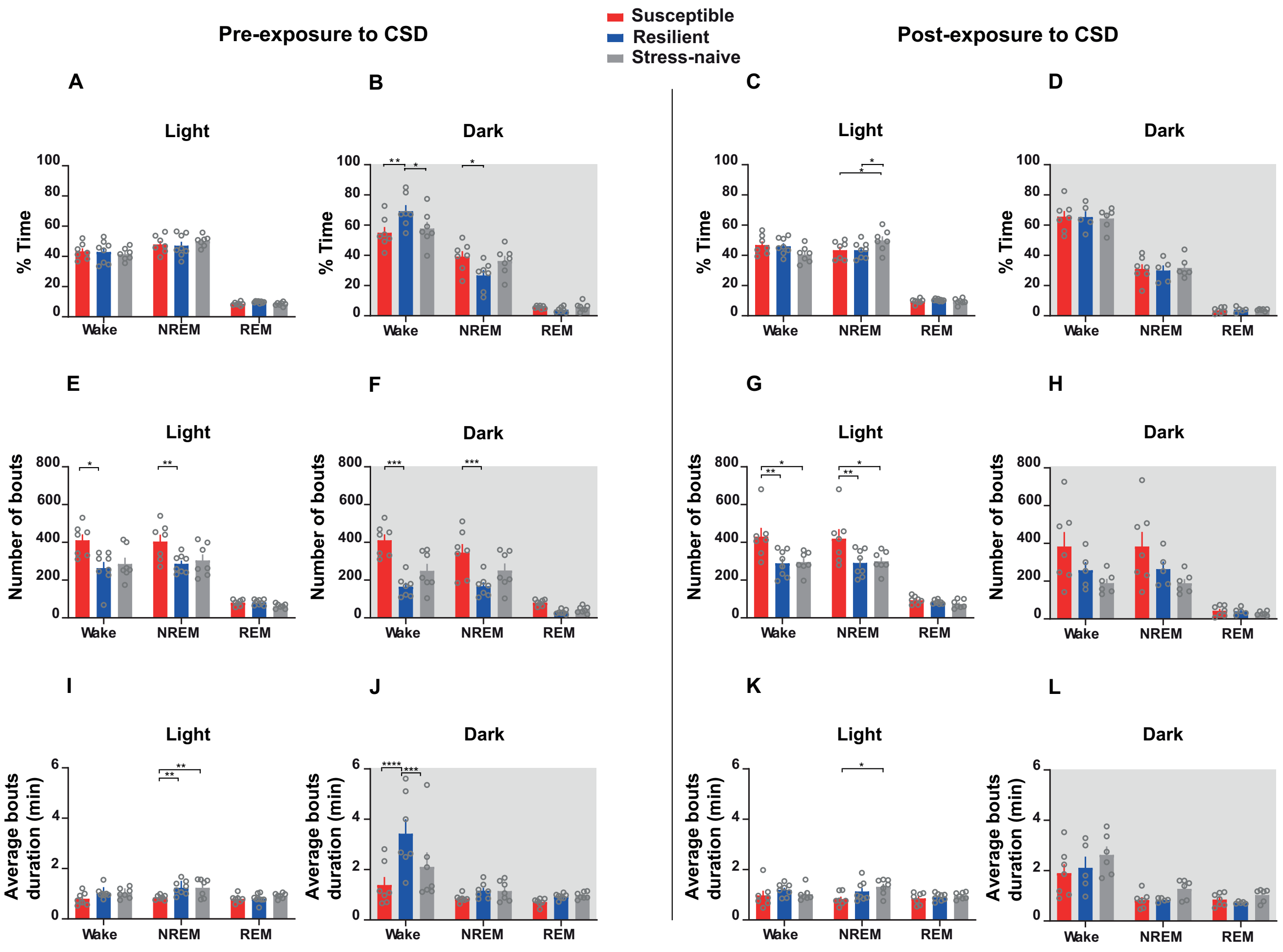

Supplementary Figure 2.

Differences in sleep-wake states between the phenotypes pre- and post-CSD. A two-way ANOVA was performed with between-factor 'phenotype' (Susceptible vs. Resilient vs. Stress-naïve) and within-factor 'Vigilance state' (Wake vs. NREM vs. REM). **(A-D)** Average percent time spent in vigilance states pre- and post-CSD during the light and dark. *Pre-CSD stress:* **(A)** No difference in the time spent in the vigilance states between phenotypes during the light. There was only a significant effect of vigilance state ($F_{2,57}$= 422.7, p<0.0001). **(B)** Resilient mice spend more time in wake during the dark relative to susceptible and stress-naïve mice (p <0.01 and p <0.05 respectively). Moreover, the resilient mice spent less time in NREM relative to the susceptible mice (p <0.05). There was a significant effect of vigilance states ($F_{2,54}$ = 232.3, p<0.0001) during the dark and a significant interaction effect between the vigilance state and phenotype ($F_{4,54}$ =5.07, p<0.001). *Post-CSD stress:* **(C)** Susceptible and resilient mice spent less time in NREM sleep compared to stress-naïve mice during the light (p <0.05 and p <0.05 respectively). Additionally, the stress-naïve mice spent less time in wake relative to susceptible (p = 0.052). There was a significant effect of vigilance states ($F_{2,36}$ = 218.3, p<0.0001) and a significant interaction effect between the vigilance state and phenotype ($F_{2,36}$ = 5.24, p<0.05). **(D)** There was only a significant effect of vigilance state ($F_{2,45}$ = 367.9, p<0.0001) during the dark. **(E-H)** Average number of bouts of vigilance states pre- and post-CSD during the light and dark. *Pre-CSD*

stress: **(E)** The number of wake and NREM bouts in susceptible mice was greater than in resilient mice (wake: $p<0.05$; NREM: $p<0.01$ respectively).There was a significant effect of vigilance state ($F_{2,57} = 111.8$, $p<0.0001$) and of phenotype ($F_{2,57} = 10.90$, $p<0.01$) during the light. **(F)** The number of wake and NREM bouts was greater in susceptible mice relative to resilient mice ($p<0.001$ and $p <0.001$ respectively). There was a significant effect of vigilance state ($F_{2,51} = 45.03$, $p<0.0001$), phenotype ($F_{2,51} = 13.22$, $p<0.0001$) in the dark. *Post-CSD stress:* **(G)** The number of wake and NREM bouts in susceptible mice was greater than in resilient and stress-naïve mice (wake: $p<0.01$ and $p<0.05$ respectively; NREM: $p<0.01$ and $p<0.05$ respectively) during the light phase. There was a significant effect of vigilance state ($F_{2,57} = 78.94$ $p<0.0001$) and phenotype ($F_{2,57} = 8.94$, $p<0.001$). **(H)** There was a significant effect of vigilance state ($F_{2,45} = 41.82$, $p<0.0001$) and of phenotype ($F_{2,45} = 3.86$, $p<0.05$). **(I-L)** Average duration of bouts of vigilance states pre- and post-CSD during the light and dark. *Pre-CSD stress:* **(I)** Susceptible mice displayed shorter NREM average duration relative to resilient and stress-naïve mice during the light phase ($p<0.01$and $p<0.01$ respectively). There was a significant effect of vigilance state ($F_{2,57} = 8.01$, $p<0.001$) and phenotype ($F_{2,57} = 7.404$, $p<0.01$). **(J)** Resilient mice displayed longer wake bouts relative to susceptible and stress-naïve mice ($p<0.0001$ and $p<0.001$ respectively). There was a significant effect of vigilance state ($F_{2,54} = 20.39$ $p<0.0001$) and phenotype ($F_{2,54} = 6.055$, $p<0.01$) and an interaction effect between the vigilance state and phenotype ($F_{4,54} =3.175$, $p< 0.05$) during the dark phase. *Post-CSD stress:* **(K)** Susceptible mice displayed shorter average duration of NREM bouts relative to stress-naïve mice ($p<0.01$). There was a significant effect of phenotype ($F_{2,57} = 3.31$, $p<0.05$) during the light phase. **(L)** During the dark phase, there was a significant effect of vigilance state ($F_{2,45} = 29.72$, $p<0.0001$) and of phenotype ($F_{2,45} = 3.49$, $p<0.05$). Post-hoc analyses were performed using Tukey's multiple comparisons. Data are averaged across 12-h intervals (Light and Dark) (mean ± sem). *n*= 7-8 for each group.

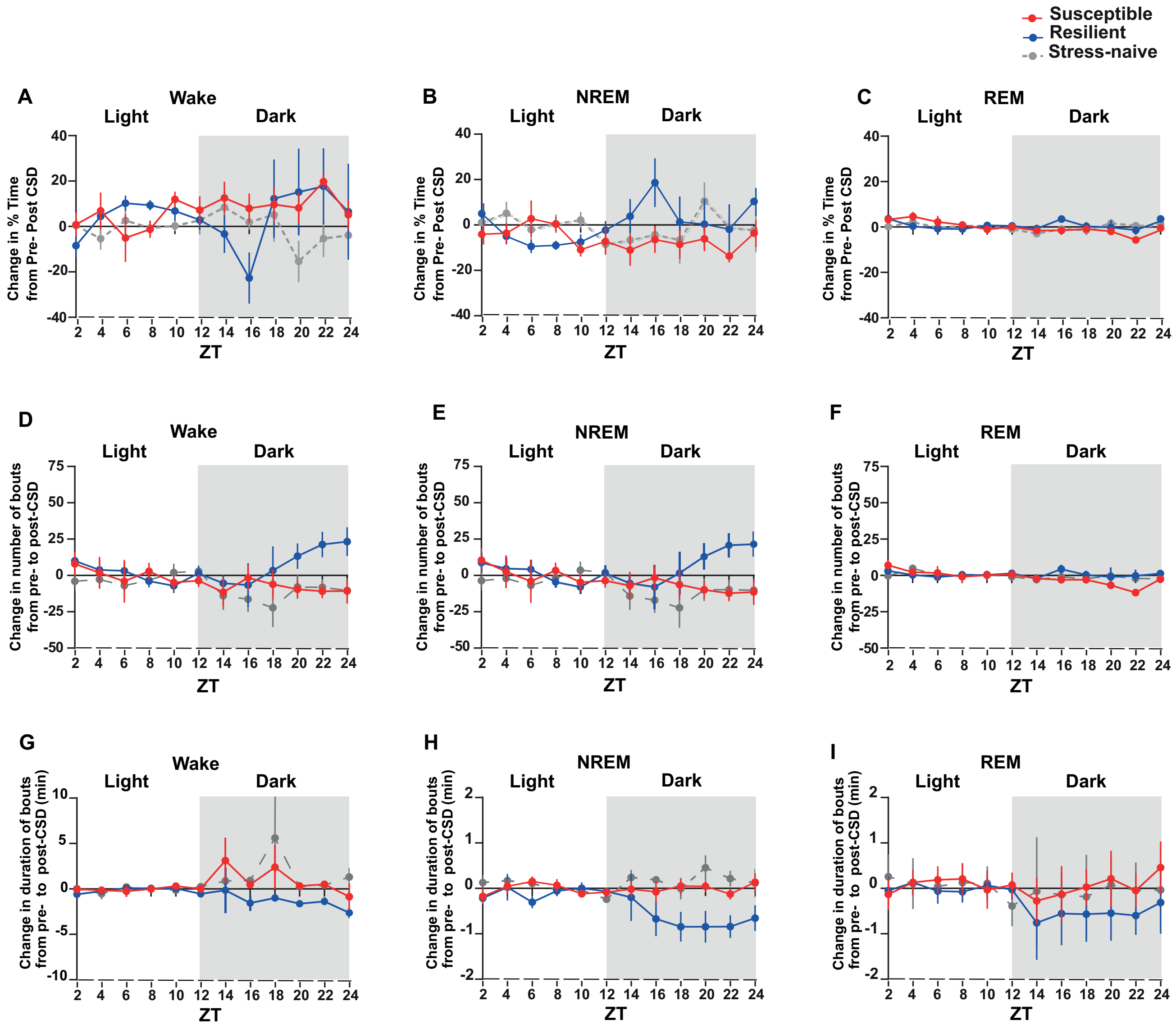

Supplementary Figure 3.

(A-C) Difference in the percentage of time in NREM, REM and wake, respectively, between post- and pre-stress exposure. Mixed-model ANOVA was performed for the light (6X2h) and dark (6x2h) between susceptible and resilient mice. **(B)** Resilient mice spent more time in NREM sleep in the dark phase than the susceptible mice, post-CSD relative to pre-CSD ($F_{1,10} = 5.24$, $p<0.05$) and **(C)** more time in REM sleep in the dark phase than the susceptible mice, post-CSD relative to pre-CSD ($F_{1,9} = 17.77$, $p<0.001$). **(D-F)** Difference in the number of NREM, REM and wake bouts between post- and pre-stress exposure. **(D-E)**. A mixed-model two-way ANOVA was performed for each panel for the first and second half of the dark phase (3x2h) between all three phenotypes. The resilient mice displayed an increase in the number of bouts of Wake and NREM, post-CSD relative to pre-CSD, compared to susceptible and stress naïve mice, in the second half of the dark (wake: $F_{2,14} = 9.969$, $p<0.01$, NREM: $F_{2,14} = 9.771$, $p<0.01$ respectively). **(G-I)** Difference of the average duration of

NREM, REM and wake bouts, respectively, between post-and pre-CSD stress exposure. **(H)** Resilient mice displayed a decrease in the average duration of NREM bouts, post-CSD relative to pre-CSD, compared to susceptible and stress naïve mice in the second half of the dark phase ($F_{2,18} = 3.87$, $p<0.05$). Data are averaged across 2-h intervals (mean ± sem). *n*= 7-8 for each group.

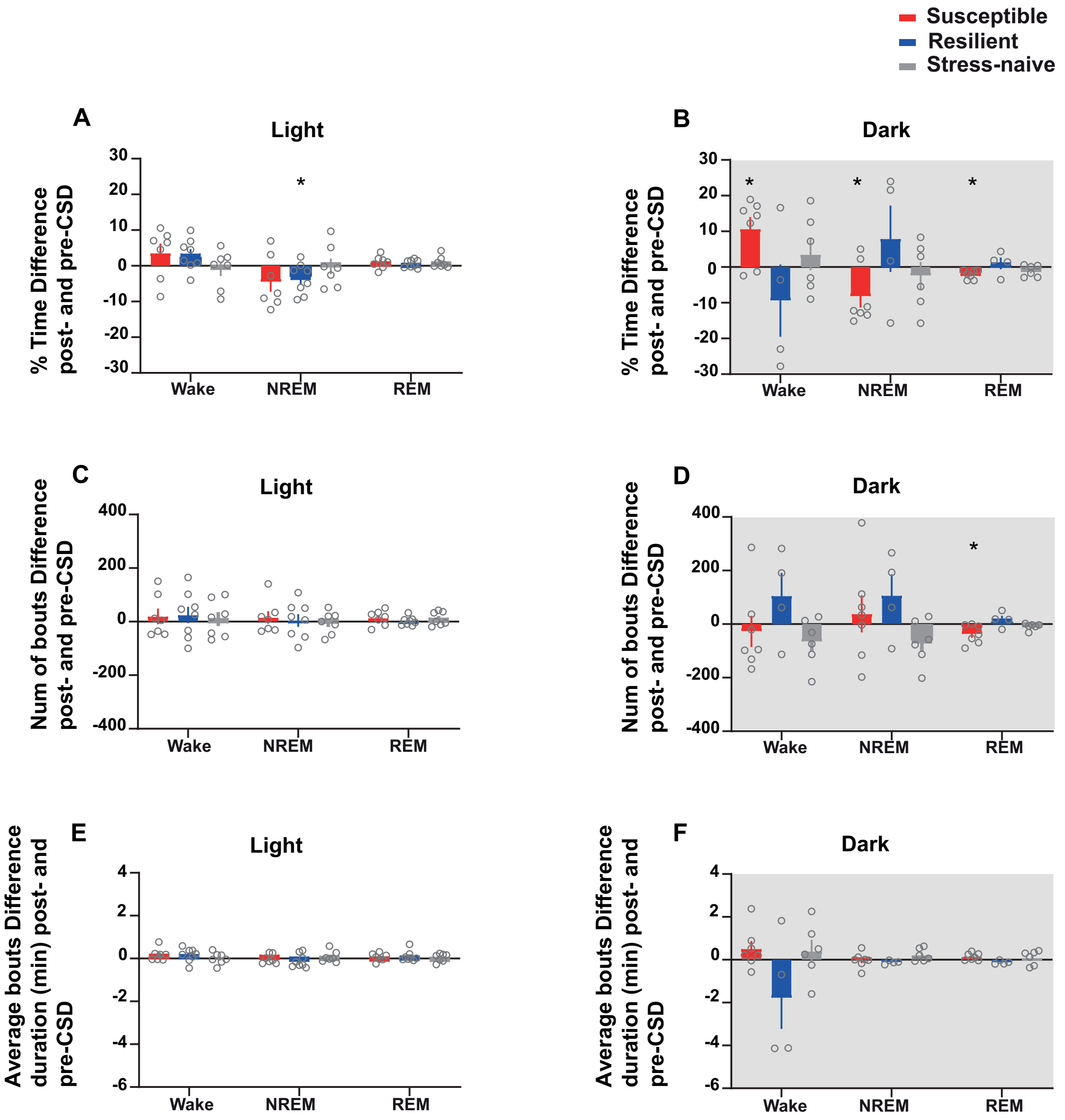

Supplementary Figure 4.

Difference in %Time, Number of bouts and Average bout duration of wake and sleep states between post- and pre-stress exposure. **(A)** In the light phase, resilient mice spent less time in NREM sleep post-CSD relative to pre-CSD ($p<0.05$). **(B)** In the dark phase, susceptible mice spent more time awake and less time in NREM and REM sleep post-CSD relative to pre-CSD ($p<0.05$ for all three tests). **(C)** No change in the number of bouts of wake and sleep states was observed post-CSD relative to pre-CSD in the light phase, **(D)** Susceptible mice exhibited lower number of REM bouts post-CSD relative to pre-CSD ($p<0.05$) in the dark phase. **(E-F)** There was no change in the average duration of wake and sleep bouts in all phenotypes either in the light or the dark phase. Data are averaged across 12-h intervals (mean ± sem). One sample t-tests were performed on the differences of each sleep/wake parameter of each phenotype. *n*= 4-7 for each group.

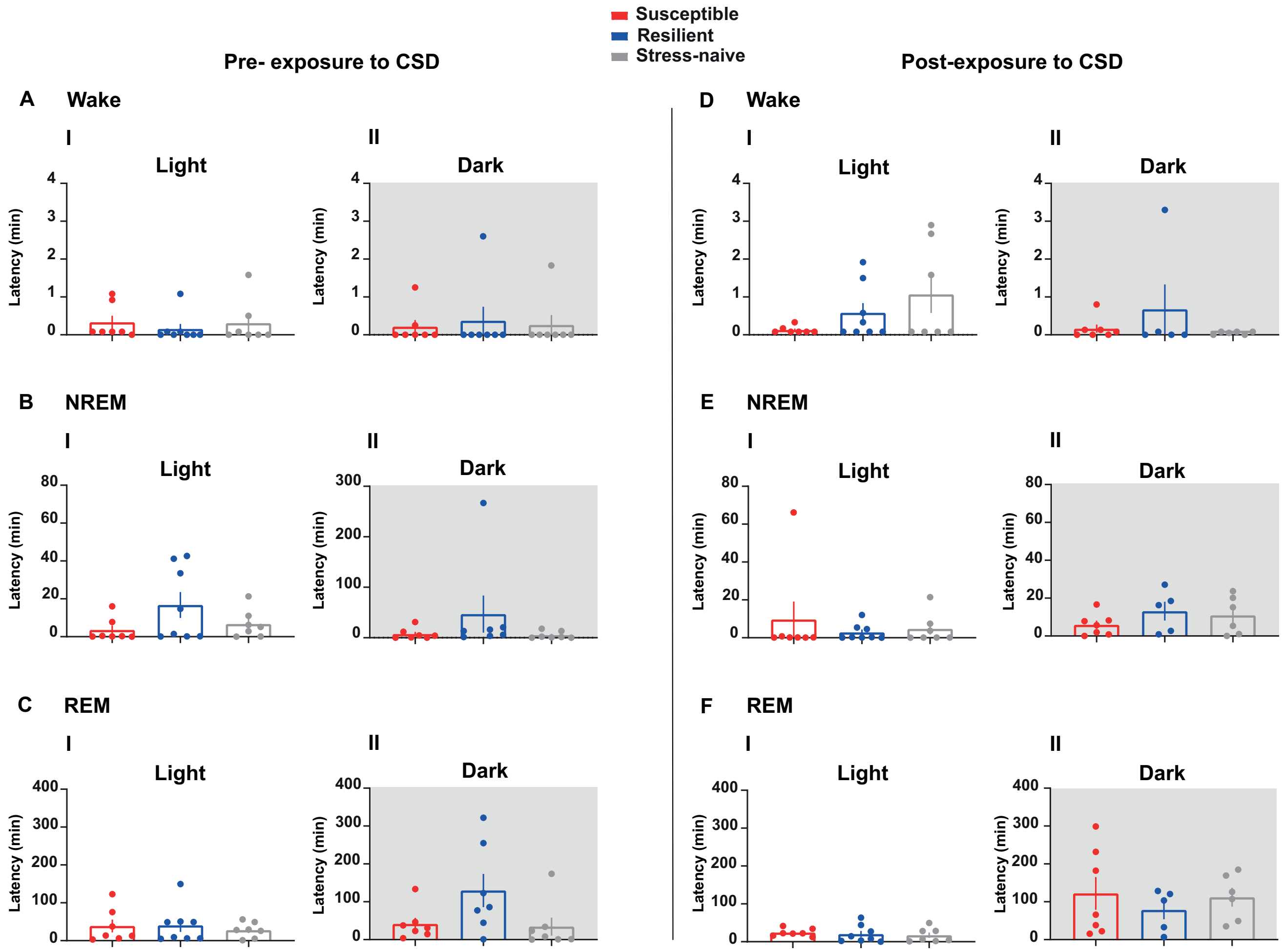

Supplementary Figure 5.

Bout latency of the different vigilance states during the (I) light (12-h) and (II) dark phase(12-h) pre- **(AI-CII)** and post-CSD stress **(DI-FII)**. **Table 1** includes the results of a one-way ANOVA performed for each sub-figure **(AI-FII)**. **Table A** displays the setup of a mixed-model ANOVA used to analyze across conditions to determine between-factor 'phenotype' (Susceptible vs. Resilient vs. Stress-naïve) and within-factor 'stress' and **Table 2** includes the results of the analysis. **(BI& EI)** During the light phase, there was a significant reduction of the latency of NREM in resilient mice post-CSD relative to pre-CSD (Mixed-model: $F_{1,18} = 4.62$, $p < 0.05$, post-hoc analysis using Sidak's multiple comparisons test for the resilient phenotype: $p < 0.05$). **Table B** displays the setup of a mixed-model ANOVA used to analyze across phases with 'phenotype' (Susceptible vs. Resilient vs. Stress-naïve) as the between-factor and 'phase' as the within-factor. **(EI-EII)** Post-CSD, there was a significant increase in the latency of NREM sleep in the dark relative to the light phase (Mixed-model: $F_{1,14} = 10.25$, $p < 0.001$)**.** **(FI-FII)** Additionally, post-CSD, there was a significant increase in the latency of REM sleep in the dark relative to the light phase (Mixed-model: $F_{1,15} = 20.49$, $p < 0.001$) specifically for the susceptible and the stress-naïve mice (Post-hoc analysis using Sidak's multiple comparisons test : $p < 0.05$) **(Table 3).** Values are expressed as mean ± sem. *n*= 7-8 for each group.

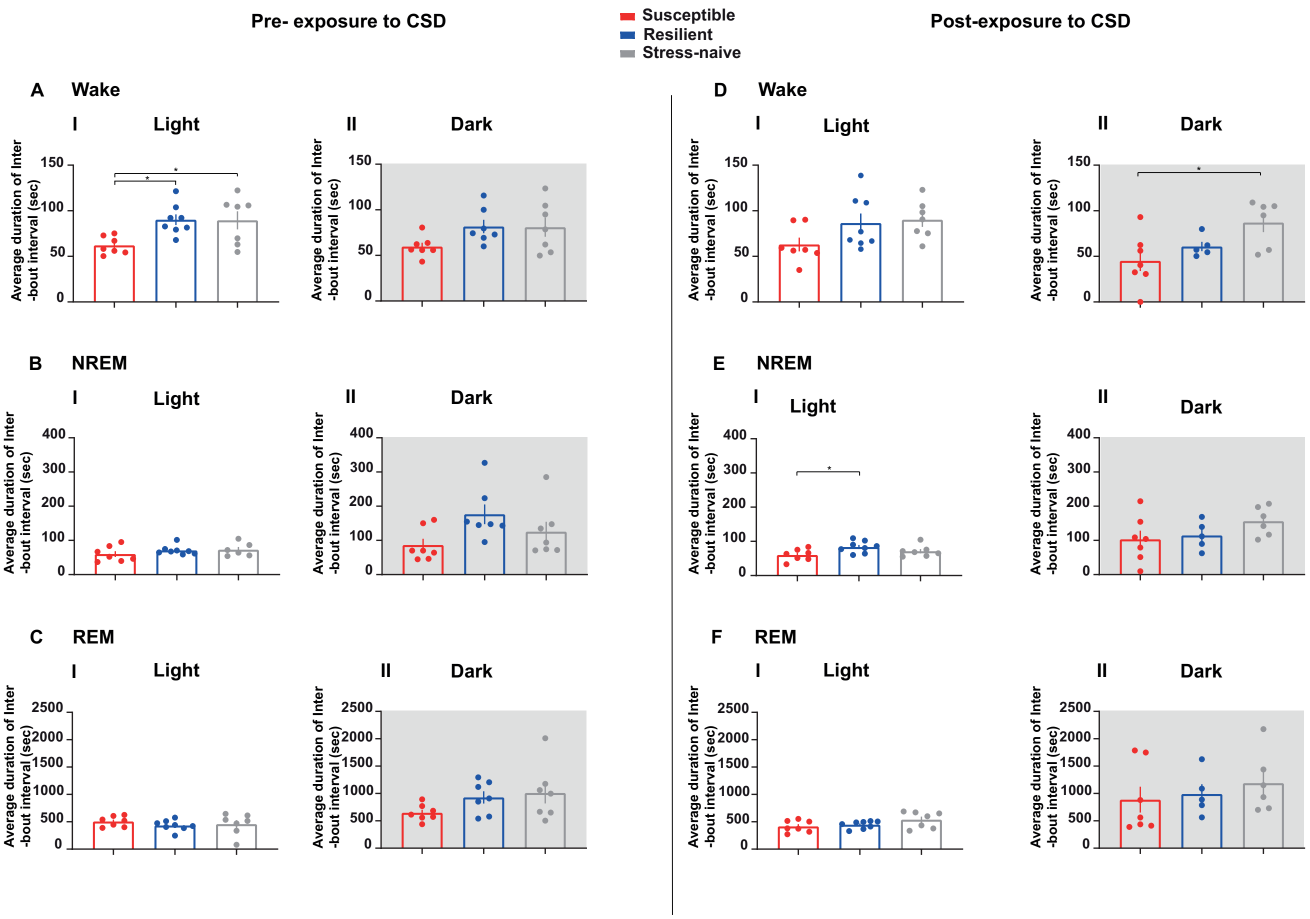

Supplementary Figure 6.

The average duration of inter-bout interval of the different vigilance states during the (I) light (12-h) and (II) dark phase (12-h) pre- **(AI-CII)** and post-CSD stress **(DI-FII)**. **Table 4** summarizes the one-way ANOVA performed for each figure **(AI-FII). (AI)** Pre-CSD: the average duration of inter-wake interval of susceptible mice was shorter in the light phase relative to the resilient and to the stress-naïve mice ($F_{2,19}$= 5.32, $p < 0.05$) and (post-hoc analysis Tukey's multiple comparisons test: $p < 0.05$ for both comparisons). **(DII)** *Post-CSD:* the average duration of inter-wake interval of susceptible mice was shorter in the dark phase relative to the stress-naïve mice ($F_{2,14}$= 4.94, $p < 0.05$, post hoc analysis using Tukey's multiple comparisons test: $p < 0.05$). **(EI)** *Post-CSD:* The average duration of inter-NREM interval of susceptible mice was shorter in the light phase relative to the resilient mice ($F_{2,19}$ = 3.586, $p < 0.05$, post hoc analysis using Tukey's multiple comparisons test: $p < 0.05$) **Table A** displays the setup of a mixed-model ANOVA used with 'phenotype' (Susceptible vs. Resilient vs. Stress-naïve) as between-factor and 'stress' as the within-factor. **Table 5** includes the results of the analysis. Susceptible mice had shorter average duration of inter-wake interval relative to the resilient and control mice from pre- to post-CSD during both the light and dark phases ($F_{2,19}$ = 4.35, $p < 0.05$, $F_{2,18}$ = 4.82, $p < 0.05$ respectively) **(AI-DI and AII-DII)**. **Table B** displays the setup of a mixed-model ANOVA with 'phenotype' (Susceptible vs. Resilient vs. Stress-naïve) as between-factor and 'phase' as the within-factor. **Table 6** includes the results of the analysis. There was a significant difference of the average duration of inter-bout interval between the three phenotypes from light-to-dark phase (p

<0.001, except in DI-DII analysis yields $p<0.05$ for the phase effect). **(AI-AII)** Pre-CSD, there was a decrease in the average duration of inter-wake interval in the resilient and stress-naive mice from light to dark phase ($F(2,19) = 3.81$, $p<0.05$; post hoc analysis using Sidak's multiple comparisons test : $p<0.01$ and $p<0.05$ respectively). **(CI-CII)** Pre-CSD, there was an increase in the average duration of inter-REM interval from light to dark phase in resilient and stress-naive mice ($F(1,17) = 31.41$ for 'phase', $p<0.001$, 'phenotype' x 'phase' F(2,17=3.70, $p <0.05$; post hoc analysis using Sidak's multiple comparisons test: $p<0.01$ and $p<0.001$ respectively). **(DI-DII)** Moreover, post-CSD, the average duration of inter-wake interval in the susceptible mice decreased from light to dark phase ($F(1,14) = 7.35$ 'phase', $F(2,19) = 4.96$ 'phenotype', $p<0.05$ for both; post-hoc analysis using Sidak's multiple comparisons: $p<0.05$). Data are averaged across 12-h intervals (Light and Dark) (mean ± sem). *n*= 7-8 for each group.

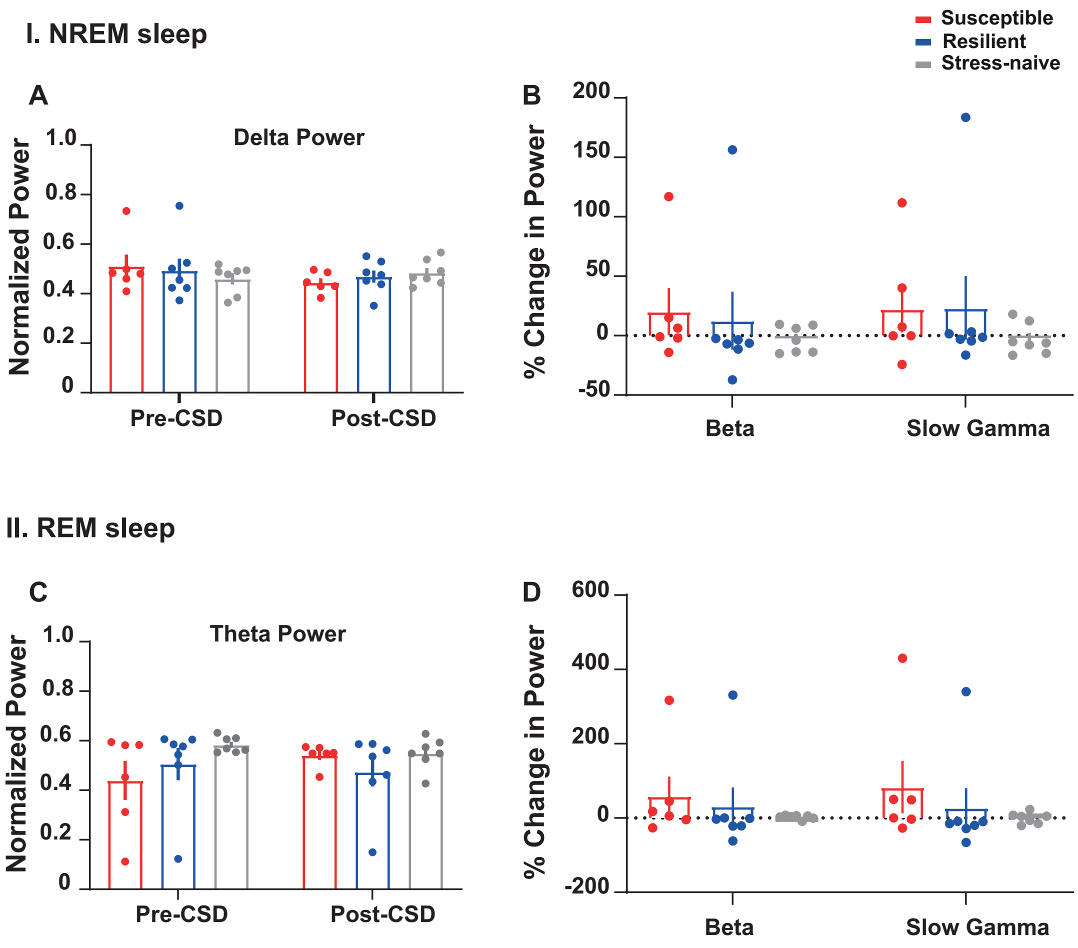

Supplementary Figure 7.

Spectral analysis of delta, theta and change in power of beta and slow gamma in NREM and REM sleep in the light. *NREM sleep:* **(A)** No change in delta power (0.5-4.5 Hz) was observed between the phenotypes either pre-CSD or post-CSD nor across pre- and post-CSD. **(B)** There was no change in beta (15-25 Hz) and slow gamma (30-45 Hz) between post- and pre-CSD in all the phenotypes. *REM sleep*: **(C)** No change in theta power (5-10 Hz) was observed between the phenotypes either pre-CSD or post-CSD nor across pre- and post-CSD. **(D)** There was no change in the beta (15-25 Hz) and slow gamma (30-45 Hz) between post- and pre-CSD in all the phenotypes. Delta and theta powers are normalized by dividing their values with the total power in the spectrum (0-45 Hz). Data are averaged across 12-h intervals, light and dark phases (mean ± sem). *n*= 6-7 for each group.

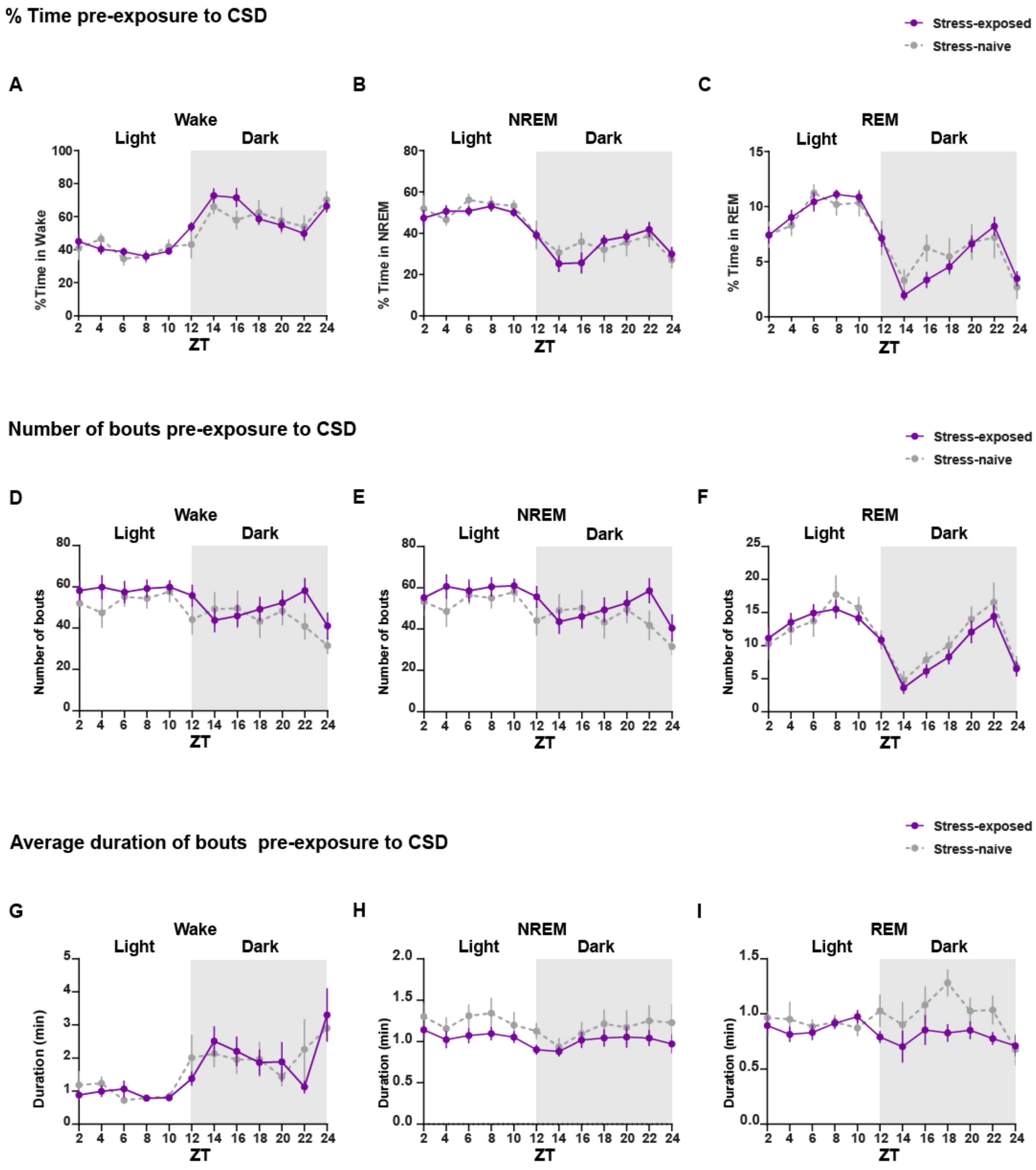

Supplementary Figure 8.

Pre-CSD stress: There was no difference between the percent time, number of bouts and average duration of the different vigilance states between stress-naïve and stress-exposed mice (combined susceptible and resilient mice vigilance states' measurements). **(A-C)** The combined percentage of

time, **(D-F)** number of bouts and **(G-I)** average duration of bouts of the three vigilance states of the resilient and susceptible (stress-exposed) mice were similar to those of the stress-naïve mice ($p>0.05$). A mixed-model ANOVA was performed for each panel with between-factor ‘phenotype’ (Susceptible vs. Resilient vs. Stress-naïve) and within-factor ‘time’ (12x 2h). Data are averaged across 2-h intervals. Values are expressed as percentage of total recording time **(A-C)** or as number of bouts **(D-F)** or as average bouts duration **(G-I)**(mean ± sem). *n*= 7-8 for each group.

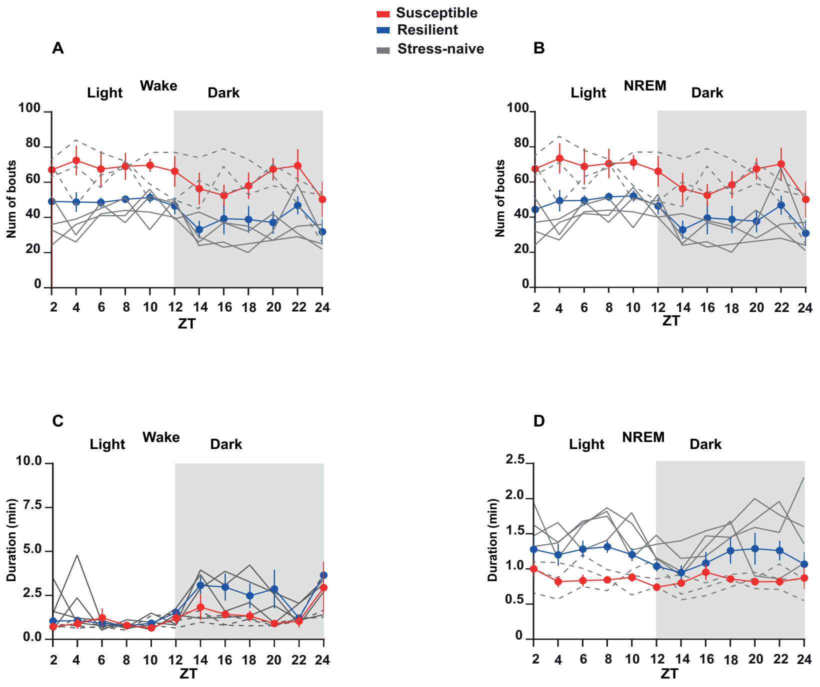

Supplementary Figure 9.

Pre-CSD: Individual observations of time series of number of bouts and average bout duration of wake and NREM of stress-naïve, showing some values similar to the average value of susceptible, and some similar to the average value of resilient mice. Wake and NREM vigilance states display the strongest separation between the susceptible and resilient mice pre-exposure to stress, due to the high NREM fragmentation in susceptible mice. Therefore, we used the wake and NREM vigilance states to display that the stress-naïve group is possibly made up of both susceptible and resilient mice pre-CSD. **(A-B)** Pre-CSD number of bouts of wake and NREM bouts respectively. **(C-D)** Pre-CSD average duration of wake and NREM bouts respectively. The dotted gray lines show the stress-naïve values close to those of susceptible mice, while the solid gray lines show the stress-naïve values close to those of resilient mice. Data are averaged across 2-h intervals (mean ± sem).

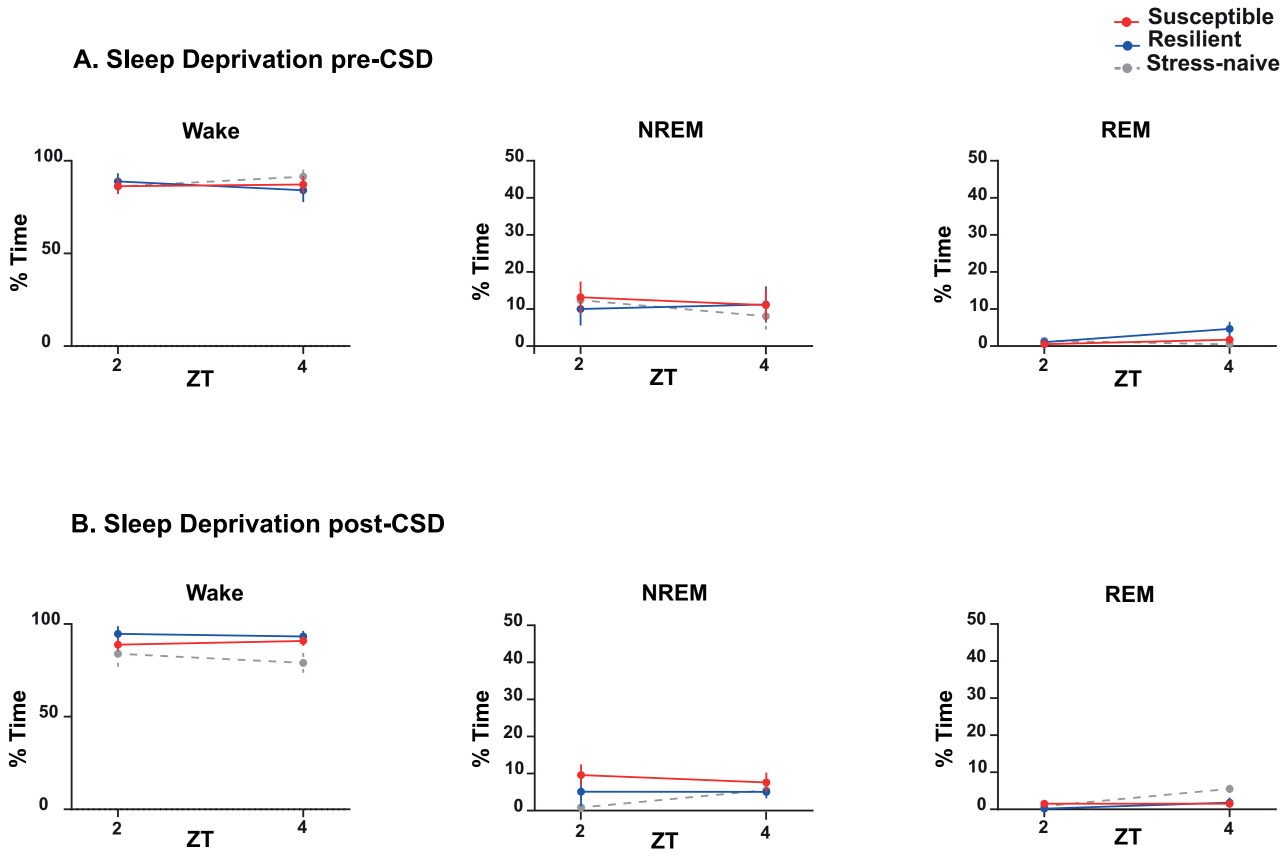

Supplementary Figure 10.

Validation of 4-h sleep deprivation using the Viewpoint platform system by sending random electric pulses in a randomized sequence to the magnet placed under the platform which pushes it up and wakes up the mice **(A)** pre-CSD and **(B)** post-CSD by scoring and analyzing the %Time of the vigilance states. Data (%Time) are averaged across 4-h intervals (mean ± sem)

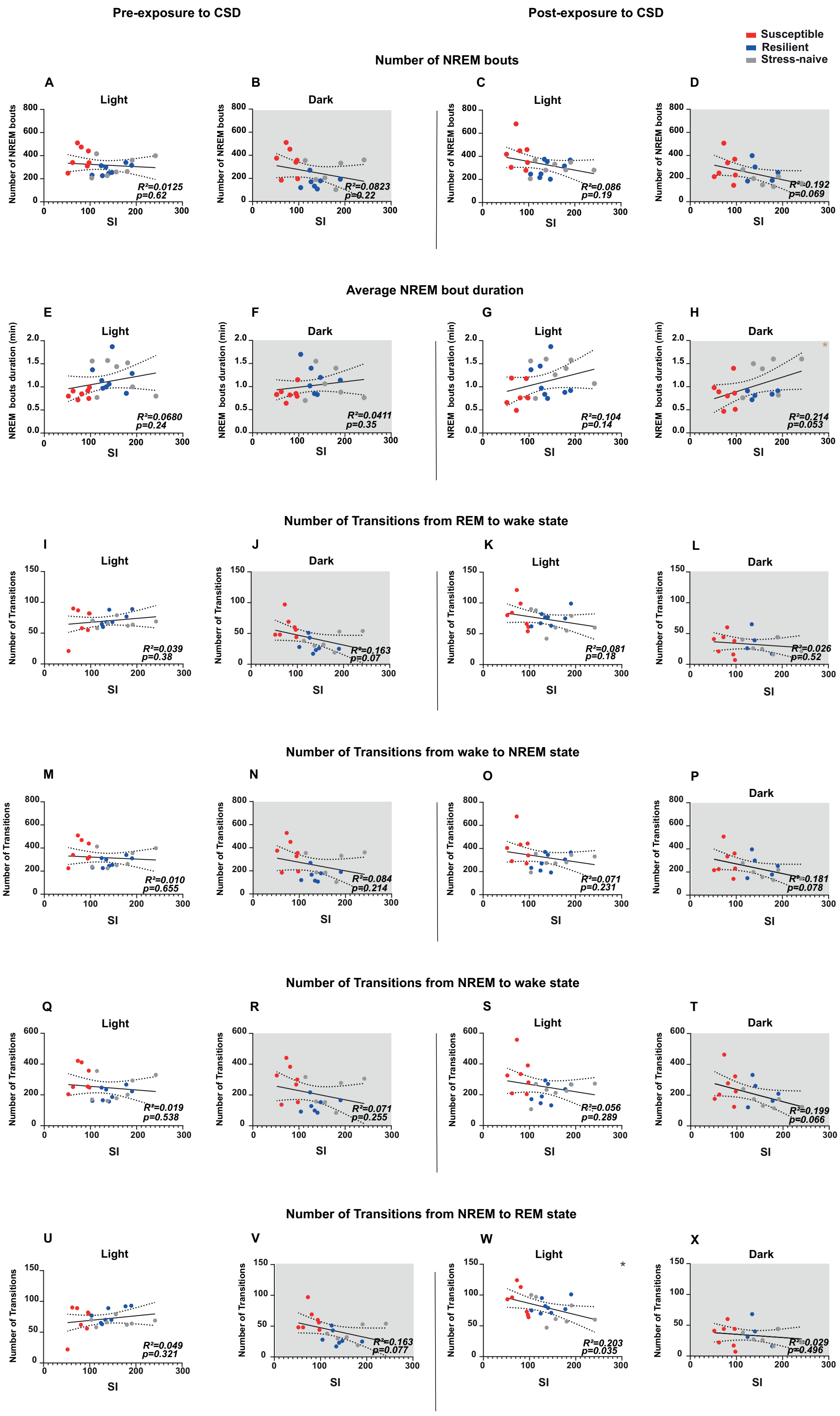

Pre-exposure to CSD
Post-exposure to CSD
Susceptible
Resilient
Stress-naive
Number of NREM bouts
A
B
C
D
Light
Dark
Light
Number of NREM bouts
R²=0.0125
p=0.62
R²=0.0823
p=0.22
R²=0.086
p=0.19
R²=0.192
p=0.069
SI
Average NREM bout duration
E
F
G
H
Light
Dark
Light
Dark
NREM bouts duration (min)
R²=0.0680
p=0.24
R²=0.0411
p=0.35
R²=0.104
p=0.14
R²=0.214
p=0.053
Number of Transitions from REM to wake state
I
J
K
L
Light
Dark
Light
Dark
Number of Transitions
R²=0.039
p=0.38
R²=0.163
p=0.07
R²=0.081
p=0.18
R²=0.026
p=0.52
Number of Transitions from wake to NREM state
M
N
O
P
Dark
R²=0.010
p=0.655
R²=0.084
p=0.214
R²=0.071
p=0.231
R²=0.181
p=0.078
Number of Transitions from NREM to wake state
Q
R
S
T
Light
Light
Dark
R²=0.019
p=0.538
R²=0.071
p=0.255
R²=0.056
p=0.289
R²=0.199
p=0.066
Number of Transitions from NREM to REM state
U
V
W
X
Light
Light
Dark
R²=0.049
p=0.321
R²=0.163
p=0.077
R²=0.203
p=0.035
R²=0.029
p=0.496

Supplementary Figure 11.

Correlation analysis conducted between SI score and **(A-D)** number of NREM bouts, **(E-H)** average duration of NREM bouts, **(I-L)** number of transitions between REM to wake, **(M-P)** number of transitions between wake to NREM, **(Q-T)** number of transitions between NREM to wake and **(U-X)** number of transitions between NREM to REM pre- and post-CSD. *Pre-CSD stress:* poor correlation between sleep parameters and SI scores. *Post CSD stress*: **(W)** Number of transitions from NREM to REM in the light are correlated with SI scores. **(A-X)** Data are averaged across 12-h intervals (either light or dark phase).

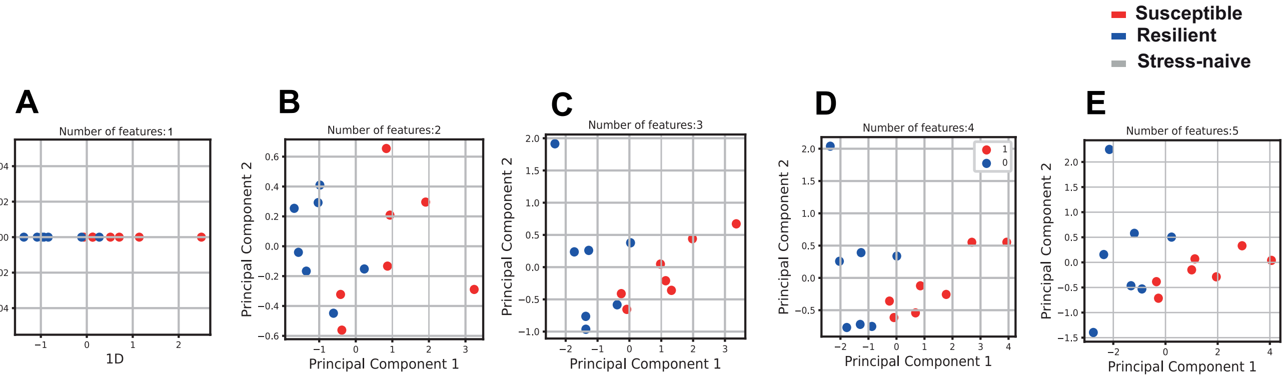

Supplementary Figure 12.

(A-E) Principal component analysis was performed to demonstrate the separation of the two phenotypes "Susceptible" and "Resilient" based on their pre-sleep features. The clusters of susceptible and resilient are separable with 2, 3, 4 and 5 top features ranked using SelectKBest algorithm. Most optimal separation of the 2 clusters occurs with 4 top features.

1.2 Supplementary Tables

Table A. Setup of the Mixed-model ANOVA performed to compare between pre- and post-CSD conditions in a particular phase (Light or Dark).

	Light	Dark
**Wake**	*AI-DI*	*AII-DII*
**NREM**	*BI-EI*	*BII- EII*
**REM**	*CI-FI*	*CII-FII*

Table B. Setup of the Mixed-model ANOVA to compare between Light and Dark phases either pre-CSD or post-CSD.

	**Pre**	**Post**
**Wake**	*AI&II*	*DI&II*
**NREM**	*BI&II*	*EI&II*
**REM**	*CI&II*	*FI&II*

Table 1. Results of one-way ANOVA on bout latency during the light and dark phase, pre- and post-CSD in between all three phenotypes.

	Pre-exposure to stress		Post-exposure to stress	
	Light	Dark	Light	Dark
Wake	*ns*	*ns*	*ns*	*ns*
NREM	*ns*	*ns*	*ns*	*ns*
REM	*ns*	*ns*	*ns*	*ns*

Table 2. Results of the Mixed-model ANOVA to compare between the bouts latencies between pre- and post-CSD conditions in a particular phase (Light or Dark).

	Light	Dark
**Wake**	*ns*	*ns*
**NREM**	$p<0.05$ *(stress)*	*ns*
**REM**	*ns*	*ns*

Table 3. Mixed-model ANOVA performed to compare bouts latencies between Light and Dark phases, either pre-CSD or post-CSD.

	**Pre**	**Post**
**Wake**	*ns*	*ns*
**NREM**	*ns*	$p<0.01$ *(phase)*
**REM**	*ns*	$p<0.001$ *(phase)*

Table 4. Results of one-way ANOVA on inter-bout interval average duration during the light and dark phases, pre- and post-CSD in between all three phenotypes.

	Pre-exposure to stress		Post-exposure to stress	
	Light	Dark	Light	Dark
Wake	*	*ns*	*ns*	*
NREM	*ns*	*ns*	*	*ns*
REM	*ns*	*ns*	*ns*	*ns*

Table 5. Results of mixed-model ANOVA performed to compare between the inter-bout interval average duration between pre- and post-CSD conditions in a particular phase (Light or Dark).

	Light	Dark
**Wake**	*$p<0.05$ phenotype*	*$p<0.05$ phenotype*
**NREM**	*ns*	*ns*
**REM**	*ns*	*ns*

Table 6. Results of mixed-model ANOVA performed to compare between the inter-bout interval average duration between Light and Dark phases, either pre-CSD or post-CSD.

	**Pre**	**Post**
**Wake**	*$p<0.001$ phase, $p<0.05$ phenotype*	*$p<0.05$ phase, $p<0.05$ phenotype*
**NREM**	*$p<0.001$ phase*	*$p<0.001$ phase*
**REM**	*$p<0.001$ phase, $p<0.05$ phase*phenotype*	*$p<0.001$ phase*

Table 7. Ascending ordering of the pre-CSD sleep features based on the strength of their relationship with the output of susceptibility to stress. Ranking was performed using the F statistical test (ANOVA F-value) via SelectKBest algorithm.

**wake-Lat_D**	0.141
**REM-W_L**	0.208
**NREM_Lat_L**	0.283
**wake-Lat_L**	0.386
**NREM-REM_L**	0.415
**REM-Lat_L**	0.976
**NREM-Lat_D**	1.131
**REM-Int_L**	1.312
**REM-Lat_D**	3.552
**NREM-Int_L**	3.882
**REM-Int_D**	5.039
**NREM-dur_D**	5.620
**NREM-wake_L**	6.557
**Wake-Int_D**	6.699
**NREM-Int_D**	6.961
**NREM_Numbouts_L**	8.032
**Wake_NREM_L**	9.288
**NREM-dur_L**	10.139

**Wake_NREM_D**	10.979
**NREM-W_D**	12.118
**Wake-Int_L**	12.330
**NREM_Numbouts_D**	12.460
**REM-W_D**	12.995
**NREM_REM-D**	12.995
